# Qualitative Analysis of Ventilation Position and Dimension Effects on Compartment Fire Dynamics: An Experimental and Numerical Approach

**DOI:** 10.1007/s10694-025-01747-5

**Published:** 2025-05-29

**Authors:** Mohamed Beshir, Yu Wang, Antonio Cicione, Michal krajcovic, Rory Hadden, David Rush

**Affiliations:** 1 Department of Civil and Environmental Engineering, Carleton University, 1125 Colonel By Drive, Ottawa, ON K1S 5B6 Canada; 2 Center for Fire Safety Engineering, School of Engineering, University of Edinburgh, Edinburgh, EH9 3JL UK; 3 State Key Laboratory of Fire Science, University of Science and Technology of China, Hefei, 230026 People’s Republic of China; 4 Kindling, Duxbury, MA USA; 5 Hydrock Consultants Ltd, Edinburgh, UK

**Keywords:** FDS, Informal settlement dwellings, Fire dynamics, Experiments, Ventilation condition, Ventilation factor

## Abstract

Informal settlements, where over 1 billion people live globally, are extremely vulnerable to fire events. Thermally thin steel-clad timber-framed homes found in South African informal settlements are a prime example of this. In this paper, we explore, through six full-scale laboratory experiments and modelling, the influence of opening locations, areas, and aspect ratios, on the fire dynamics of thermally thin and leaky compartments. It was found that having the window on the same wall as the door produced the highest heat fluxes opposite the door (13 kW/m^2^). Having the window opposite the door on the back wall, created a crossflow scenario which produced slightly higher fluxes opposite the door (10–11 kW/m^2^) compared to when the windows were on a side wall (7–9 kW/m^2^). Increasing the opening area by including another equally sized window, or by doubling the window width or height, slightly reduced the heat fluxes opposite the door and window, in general slightly increased the time to flashover, and significantly increased the heat release rate required for flashover. The work presented within this paper adds to the growing body of knowledge around informal settlement dwelling fire dynamics which can be used by engineers and urban planners in understanding and mitigating urban conflagrations within these communities.

**Supplementary Information:**

The online version contains supplementary material available at 10.1007/s10694-025-01747-5.

## Introduction

Urbanization, whether driven by conflict, climate change, or economics, is a world-wide challenge, with housing, infrastructure, and safety, all being pressurised [1]. In the past few decades, most urban growth has occurred within the Global South (GS). It is estimated that each year the urban areas in GS, more specifically Low/Middle Income Countries (LMIC), grow by 70 million people [2]. With these increases, authorities struggle to keep up with the housing demand. This leads to Informal Settlements (ISs) being established by the urban vulnerable and has resulted in now more than one billion people currently living in ISs across the globe. This number is ever increasing, with IS residents increased by around 213 million people since 1990–2015.

In South Africa, for example, it is estimated that up to 33% of the country’s population now live in ISs, and in Cape Town alone, the number of IS dwellings (ISDs) is estimated to have increased from 28,000 in 1993 to 220,000 in 2011 [3]. In Brazil statistical studies showed that there are approximately 11 million people living in ISs which is about 6% of the total country’s population [4].

Inhabitants of ISs are often forced to use readily available materials (steel sheet, timber, plastics, cardboard, etc.) to build their homes, which are not as robust as those that would be constructed under formal building codes. This leaves homes and residents vulnerable to hazards such as fires, examples of which can be seen all round the world, such as: Imizamo Yethu, Western Cape, South Africa (2017) [5], Dhaka, Bangladesh (2019) [6], El Pochote, Costa Rica (2019) [7], Moria Refugee Camp, Lesbos (2020) [8], and many others [9].

Since 2017, there have been many studies (both numerical and experimental) to fill the gaps of knowledge for these fires and to understand the different fire spread mechanisms within ISs. Studies include: bench scale material tests (e.g., [10]); small scale experimental and numerical compartment fires to study the heat transfer and Heat release rate (HRR) onset of flashover (e.g., [11]); large outdoor full-scale compartment fire experiments to understand the difference in fire dynamics and spread between timber and steel clad ISDs (e.g., [12, 13]), and indoor lab-based large scale compartment fire experiments with steel cladding to understand the effect of different boundary conditions on the internal fire dynamics and external heat fluxes to the surroundings from the dwelling of origin (e.g., [14]).

Many of these experiments were then modelled using the Fire Dynamics Simulator (FDS) developed by NIST [15], and have been extended to also assess the effect of wind on the onset for flashover in ISDs (e.g., [16]). These studies fed into fire risk mapping using remote sensing and GIS techniques (e.g., [17]) and for fire spread modelling (e.g., [18]), and to help determine the critical separation distance between dwellings (e.g., [19, 20]). Recent experimental studies have also looked at the impact of the ventilation condition of the structure that is being spread to, to understand the impacts of leakage and changing ventilation conditions on the internal and external fire dynamics of that second structure [21].

Now, it is well known that the amount of opening area is correlated to the fire dynamics within a thermally thick compartment (e.g., [21]). The experimental and numerical research presented above also shows this to be the case for thermally thin compartments. However, what has not been fully assessed is how, or if, the location and aspect ratio of these openings impact the internal and external fire dynamics.

The ventilation conditions not only affect the amount of available fresh air in the compartment, but will also affect the amount of mixing that takes place within the compartment [22], where better mixing can highly affect the burning rate.

In his PhD, Parkes [23] conducted 34 experiments in a 1/2 height scaled ISO-9705 ceramic-lined steel compartments with dimensions of 3.6 m × 2.4 m × 1.2 m (L × W × H). The ventilation conditions were varied using full open, soffit, door, window, and small window openings, where the ventilation factor varied between 3.155, 2.4, 0.4, 0.372 and 0.186 m^5/2^. The effect of the ventilation factor was clear on the top hot gas layer and the heat fluxes on the floor. Where for the door case, the top hot gas layer average temperature was around 681 °C, while for the window (slightly smaller ventilation factor) it was 617 °C, and for the small window (around half the ventilation factor of the door case) was 779 °C. The average heat fluxes on the floor were around 65 kW/m^2^, 40 kW/m^2^ and 27 kW/m^2^, respectively. In this study, the global equivalence ratio within the compartment was linked to the presence and size of the external flaming. The study shows clearly that the higher the equivalence ratio value, the bigger the external plume. However, the study did not provide clear quantification of the exact effect of the ventilation conditions on the size of the plume, or (which might be more importantly in urban fire spread) the heat flux measurements from the openings post-flashover.

To understand the impact of the external plume Cheng and Hadjisophocleous [24] studied experimentally the radiation from a single opening, thermally-thick, compartment fire to an adjacent building via twelve full-scale compartment fire experiments. Three opening sizes were investigated, and for each window size four experiments were carried out to study the effect of different fuel loads (namely, wood cribs and a propane burner) and three separation distances between the compartment and the adjacent wall. The target wall was placed at four different separation distances 2.4 m, 3.0 m, 3.5 m, and 4.0 m, and instrumented with 10 radiometers.

For the propane-based experiments, the wider the window the taller the external plume above the window and the hotter the internal gas temperature of the compartment. For the wood crib experiments, there was no clear link between the size of the external plume and the size of the window, however, there was a clear dependency for the external plume height on the wood crib design. As expected, the design with more exposed wood surface produced the highest external plume length, due to the more pyrolyzed gases produced and hence more unburnt gases leaving the compartment.

Cheng and Hadjisophocleous’ [24] study highlighted some of the more important factors to consider when studying such scenarios (i.e., external plume radiation on target wall). However, it is still not filling the gap of knowledge required for the ISs fires. For example, it did not consider the effect of multiple openings (i.e., window and door) or the effects of leakage at construction joints that are common in informal settlement dwellings.

Beshir et al. [25] used the National Institute of Standards and Technology (NIST) Full/reduced scale enclosure (F/RSE) compartment fire experiments [26, 27] to further understand under-ventilated fires. Beshir et al. [25] qualitatively optimized Fire Dynamics Simulator (FDS) models and through parametric studies assessed (a) the influence of vertical opening locations on the external plumes, and (b) whether the results scale between full and reduced scale models. It was found that the flow field inside the compartment is easily replicated, between scales, when there was no complex internal flow between the two openings (i.e., two openings on the same wall) and when there is a crossflow (i.e., window opening opposite the door). However, it is much more complex to replicate the flow field in perpendicular flows (i.e., windows on the perpendicular side- walls). This is an observation rather than a conclusion and further data is required to perform more in-depth studies.

The aim of the study is to qualitatively understand the significance, or not, of ventilation location and size on the thermal and fluid dynamics within compartment fires and its potential expected effect of the fire spread parameters (such as external heat fluxes) when conducting large scale compartment fire experiments. Additionally, numerical models were used with the aim to replicate the burning behaviour of the compartment fire’s solid fuel (i.e., wood crib) based on optimised inputs, demonstrate the model’s ability to simulate various compartment fire phases and mirror trends in internal compartment temperatures and external plumes/heat fluxes in response to ventilation changes. The study is based on six new large scale [28] lab-based steel-clad compartment fire experiments with wood cribs as fuel load; and comparing these experiments to the Base Line (BL) case presented by Wang et al. [14].

The numerical investigation is mainly based on a FDS model for these types of compartments, where the modelling methodology and inputs were presented in details in Beshir et al. [29]. The work presented within this paper aims to aid engineers and urban planners in understanding and potentially mitigating urban conflagrations within these communities by providing qualitative understanding of the phenomena present within these complex structures and therefore allowing evidence-based decision making to occur.

## Experimental and Numerical Setups

Currently, there are very few dwellings surveys [30] conducted in ISs and there is a clear lack of quantification on the dwellings’ sizes and also the locations/sizes/numbers of the openings. However, based on some ad-hoc surveys done in the Imizamo Yethu IS in South Africa, it was found that a typical dwelling will have one door and one window. The dimensions of these dwellings vary, but usually it ranges somewhere around the ISO-9705 room dimensions [20, 28]. This study focused on moving the location of the window in relation to the door (constant location) to investigate the effect of the window location on the heat fluxes from the vertical openings to the surroundings.

### Experimental Dwelling Setup and Measurement Locations

Six dwellings with identical dimensions to ISO-9705 room (3.6 m × 2.4 m × 2.4 m) were constructed on top of 8 mm thick cement boards. These six dwellings are the final experiments to be presented from a series of 13 experiments investigating the fire dynamics and fire spread characteristics within informal settlements (other experiments can be found in [14, 31, 32]). The walls and ceiling (boundaries) were made out of galvanized steel sheets of a 0.51 mm thickness and attached together using timber frames which were 0.038 × 0.089 m in cross-section. These structures mimic dwellings found in the ISs of the Western Cape, South Africa.

The ventilation design of the cases herein was based in part on the information gathered by Beshir et al. [25] where it was concluded that placing the window on different locations on the side walls was still in need of investigation, whereas, placing the window at different locations on the back wall did not show any significant differences in results. It was also noted by Beshir et al. [32] that the effect of opening area also needed further investigation.

**Fig. 1:**
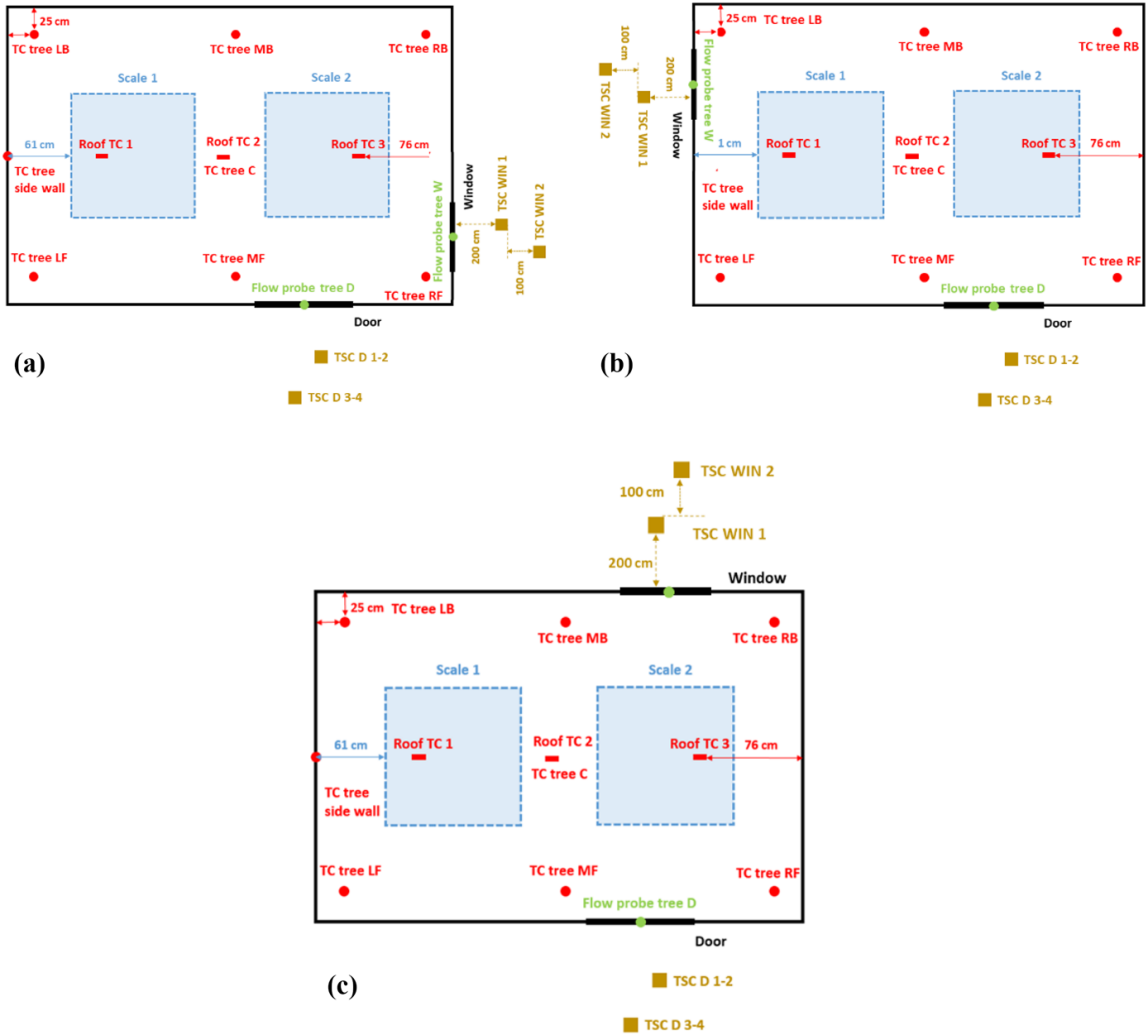
Plan view and measurement points for: **a** side right wall (SRW), **b** side left wall (SLW), and **c** back wall (BW) cases

**Fig. 2:**
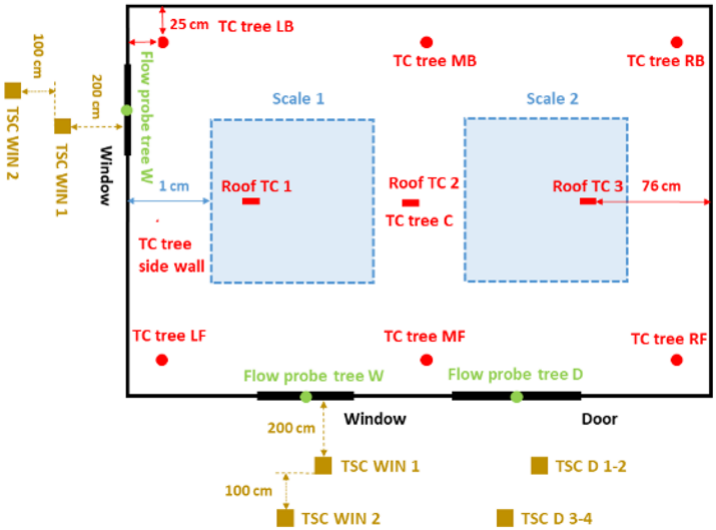
Plan view and measurement points for the DW case

**Fig. 3:**
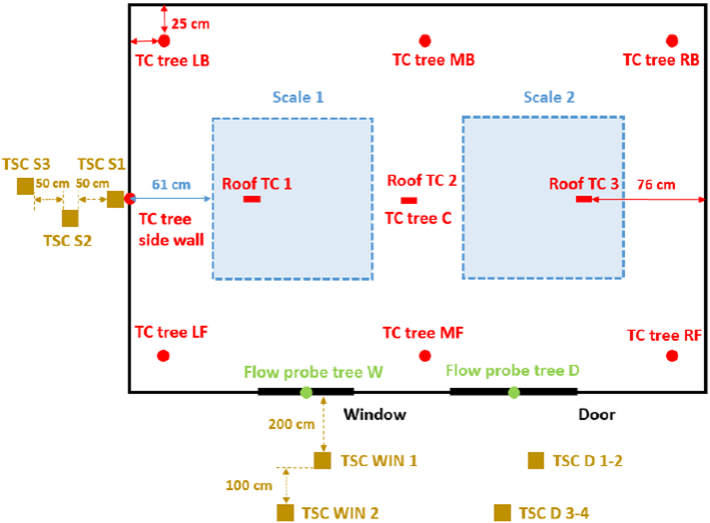
The plan of the BL experiment from [14]. NB: windows in Group 3 will either be 0.6 m (W) × 1.2 m (H), or 1.2 m (W) × 0.6 m (H)—top and centre of the window remains in the same location

**Fig. 4:**
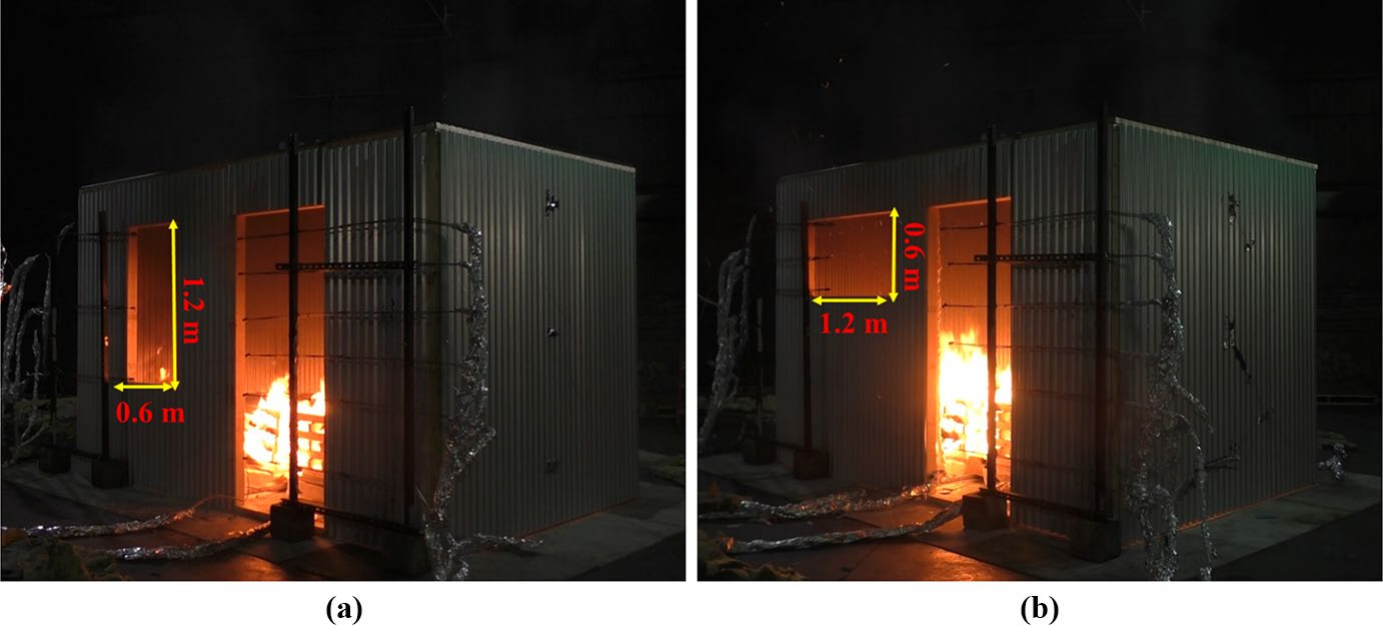
Images of **a** the VW case, **b** the HW case shortly after ignition

Every dwelling had a door with internal dimensions of 2.0 m (height) × 0.8 m (width) located 0.7 m from the bottom right-hand front corner of the structure as shown in Figs. 1 to 3. The window arrangements were then varied in three different groups and will be compared to the Base Line (BL) case presented by Wang et al. [14]. **Group 1** includes three cases where the window location is changed along different boundary walls (Side right window (SRW), Side left window (SLW) and back window (BW)). The three cases follow the same design of the BL case, but only the window location is changed as presented in Fig. 1. Where the edge of the side window for the SRW case was placed at 0.7 m from the front wall (i.e., closer to the front wall) and the edge of the side window for the SLW was placed at 0.7 m from the back wall (i.e., closer to the back wall); **Group 2** consists of one case with double windows (DW), a front window exactly the same as the BL scenario but with an additional left wall window, as presented in Figs. 2 and 3; and **Group 3** covers two cases where the window area is 0.72 m^2^ (double the size of the window in BL case) with the ventilation factor being varied by changing the aspect ratio. The aim is to study a case with shorter wider window, whereas the other case with a taller narrower window. The first case had a Vertical Window (VW) of 0.6 m × 1.2 m (W × H), and the second case had a Horizontal Window (HW) of 1.2 m × 0.6 m (W × H). The top of the windows in all cases were the kept the same—2.0 m from the finished floor. Figure 4 shows the HW and VW cases as example of how the experiments looked.

Table 1 summarize the abbreviations used to name each experiment.

**Table 1:** Experimental scenarios abbreviations

Scenario	Abbreviation
Base Line	BL
Side right window	SRW
Side left window	SLW
Back window	BW
Double windows	DW
Vertical window	VW
Horizontal window	HW

**Fig. 5:**
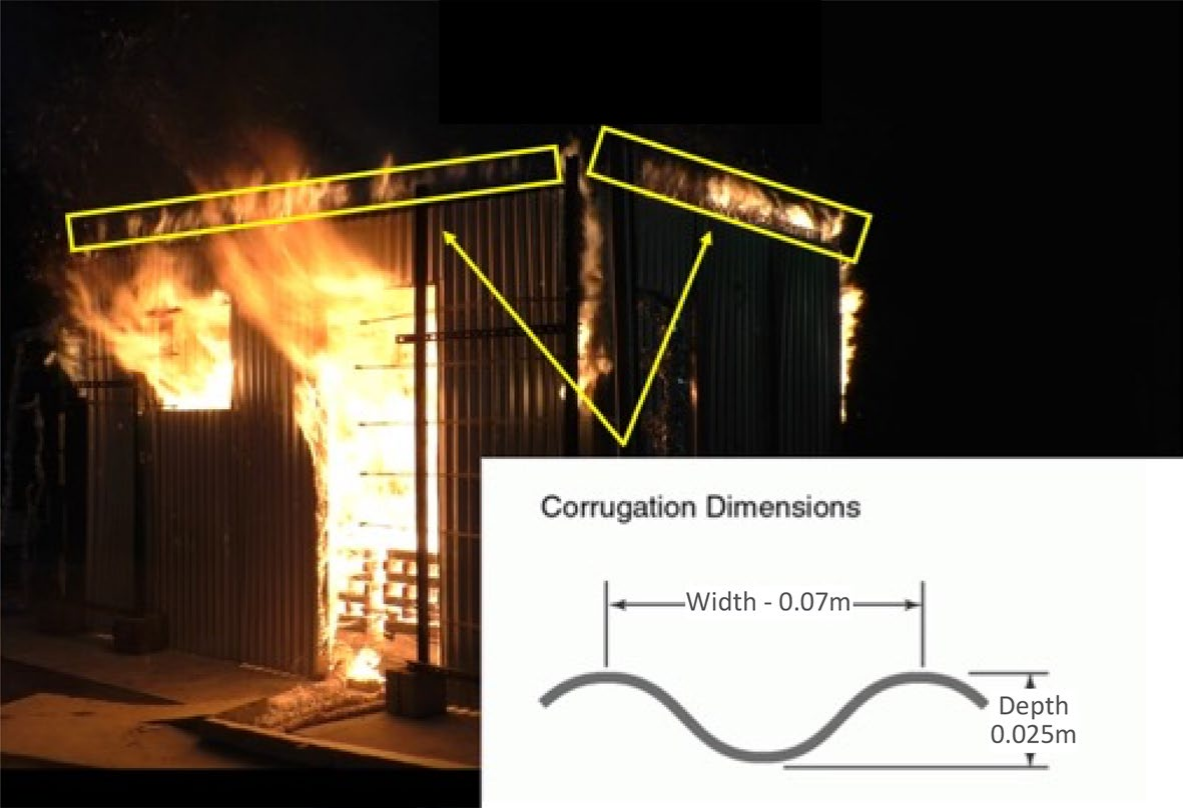
The impact of corrugation leakages on external flamming and a schematic of the corrugation’s dimensions presented (BL case)

It should be noted that none of the connection joint leakages were sealed in any of these six experiments. As presented in Fig. 5, the corrugation dimensions of the steel sheets were around 0.07 m width and 0.025 m depth. The construction gaps around these corrugations, as illustrated in Fig. 5, allows hot unburnt gases to leak out of the compartment, which turn into flames once mixed with enough air. The total leakage area is 0.000625 m^2^ per corrugation flute. A total of 51 and 34 flutes were present for the long (0.032 m^2^) and short walls (0.021 m^2^), respectively, at the tops of the walls.

The dwelling design and the fuel load were chosen based on surveys conducted in ISs in the Western Cape, SA, where the average load was found to be 410 MJ/m^2^ [30]. Therefore, two wood cribs (112 kg each) were placed at the centre of the compartment with a separation distance of 0.18 m. Each crib had 7 layers with 10 sticks in each layer. Each stick had a cross-section of 0.038 m × 0.064 m × 1.219 m and a density of 540 kg/m^3^, which gave a fuel load of approximately 438 MJ/m^2^, assuming a heat of combustion of 17.5 MJ/m^2^ [14]. The ignition source during the four tests was 8 packages of mop head strips soaked in Gasoline-87, placed in plastic bags, placed in the four corners of each crib.

**Fig. 6:**
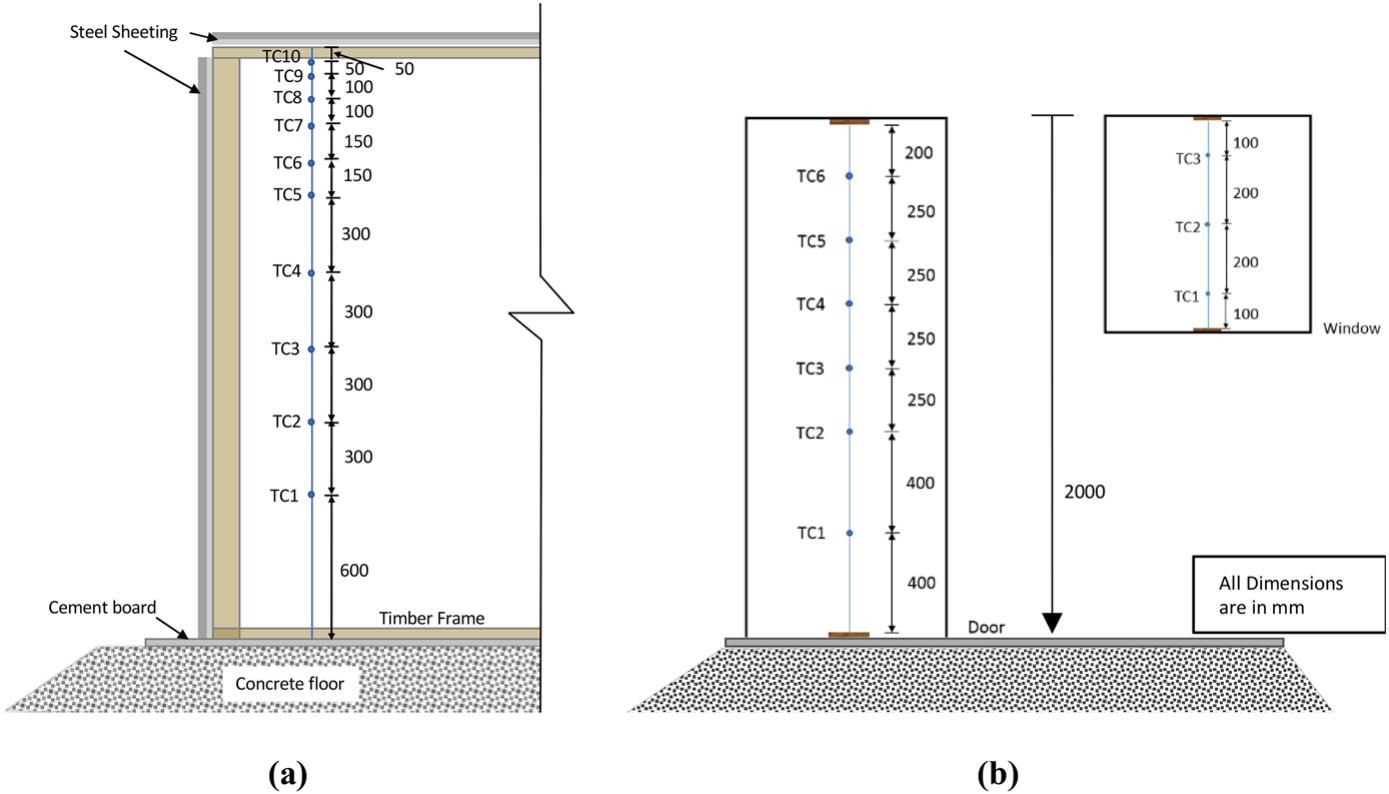
A cross-section view of the thermocouple distribution for the internal trees, and **b** the elevation views of the thermocouple and coincident flow probe locations at the openings

The tests were heavily equipped with measurement points as presented in Figs. 1 and 3. These include 1.0 m × 1.0 m scales that were placed under each crib to measure the mass loss rate of the cribs. Six thermocouple (TCs) trees were suspended from the ceiling to the floor and were named according to location (L – Left, R – Right, M – middle, F – front, B – Back). Each thermocouple tree consisted of 10 unevenly distributed Inconel sheathed Type-K thermocouples, with a 1.0 mm diameter tip, as shown in Fig. 6a, with TC1 being the lowest (600 mm from the floor) and TC10 the highest (50 mm beneath the roof). The wall temperature was measured using three thermocouples attached to the external boundary of each wall at heights of 0.4 m, 1.2 and 2.0 m. Additionally, three TCs were also attached on the external surface of the roof along the short axis centre line at the centre and at 0.76 m from both sides of the roof. Six bi-directional flow probes, with associated TCs, were placed along the vertical axis of the door to measure gas velocities in and out of the dwellings. Three additional flow probes, again with associated TCs, were placed at the vertical centre line of the window as shown in Fig. 6b (NB: for the VW case the flow probes and thermocouples TC1, and TC3, were kept 100 mm from the edge of the window opening, and TC2 was kept in the centre of the window opening). Data from flow probes can be used to calculate many associated aspects of the fire dynamics within the compartments. Within this paper, the key aspect is the estimation of the height of the neutral plane (where the flow of gases into/out of the compartment is zero). Where the “zero” flow appears to be between two measurement points (in either the experiments or modelling outlined below), the mid-height between the two points is calculated and rounded up to the nearest 5cm.

The gas concentrations (O_2_ and CO_2_) within the compartment were measured using off the shelf gas analysers with sampling points located 10 cm from the top left front and back corners and at the same location as the top window flow probe (NB: the left side is the window side). The Oxygen concentration was measured using an AO_2_ CiTicel 3 electrode oxygen cell, while CO_2_ was measured using a non-dispersive infrared sensor (NDIR) and the data was collected at 1 Hz.

The incident radiant heat fluxes outside of the compartment were measured using Thin Skin Calorimeters (TSCs) [33], where two TSCs were placed at a height of 1.6 m from the floor at distances of 2.0 and 3.0 m from the window(s) and four TSCs were placed in front of the door at two heights 1.6 m and 2.5 m at distances of 2.0 and 3.0 m from the door. To measure the radiative heat fluxes from the side walls, three TSCs were placed at a height of 1.2 m, at distances of 0.05 m, 0.5 m, and 1.0 m from the compartment (see e.g., Fig. 2a).

All point measurements data were logged at a rate of 0.2 Hz. All experiments were conducted at the burn hall of Underwriter Laboratories, Chicago, US, under a 10 MW large fire lab calorimeter hood. The gas extraction rate was controlled to be between 12 and 16 m/s during all experiments. The total Heat Release Rate (HRR) was recorded every second starting the ignition time.

The experiments were recorded using three cameras placed in front of and beside the compartment. Two cameras were set to capture the flame size out of the door and to observe the burning of the cribs inside the compartment.

The experiments were ended when; (i) the fire was burnt out, (ii) structural collapse, or partial collapse (i.e., roof or wall) or (iii) the total HRR (10MW) or gas temperature (185 ºC in exhaust flue) exceeded the maximum safety limits of the hood.

### Modelling

In this study the CFD was identified as the most suitable numerical methodology. This preference is clearly underpinned by the capacity of CFD to accurately simulate complex physical phenomena (i.e., compartment fires with solid fuel) compared to simpler methods as Zone models. While the influence of ventilation on compartment fire behaviour is relatively well established, this study focuses on investigating the specific effects on variations in ventilation size and position within the context of ISs applications/configurations. Our aim is to analyse how these variations influence the thermal and fluid dynamics within compartment fires, particularly their impact on flashover initiation and potential fire spread between ISDs. CFD can -to a good extend- capture the interplay between ventilation alterations and air entrainment, subsequently influencing the burning rate of the wood crib (i.e., fire spread between wood cribs sticks) and the fire dynamics within the compartment. On the other hand, Zone models, while offering a more simplified and computationally less expensive/intense approach, it lacks the granularity required for our specific research objectives. Modelling these compartments with Zone models will aggregate the entire wood crib into a singular heat source, thereby missing the detailed variations in the burning process of the wood crib attributable to the changes in ventilation.

Therefore, the numerical modelling of the experiments was done using the Fire Dynamics Simulator (version 6.6.5) and used the same methodology as described in Beshir et al. [29], i.e., using a time shrink factor of 5, to reduce the computational time, as well as adopting the proposed correction method for when the CFD grid cells are larger than the fuel dimensions [34].

#### Modelling Main Inputs

**Fig. 7:**
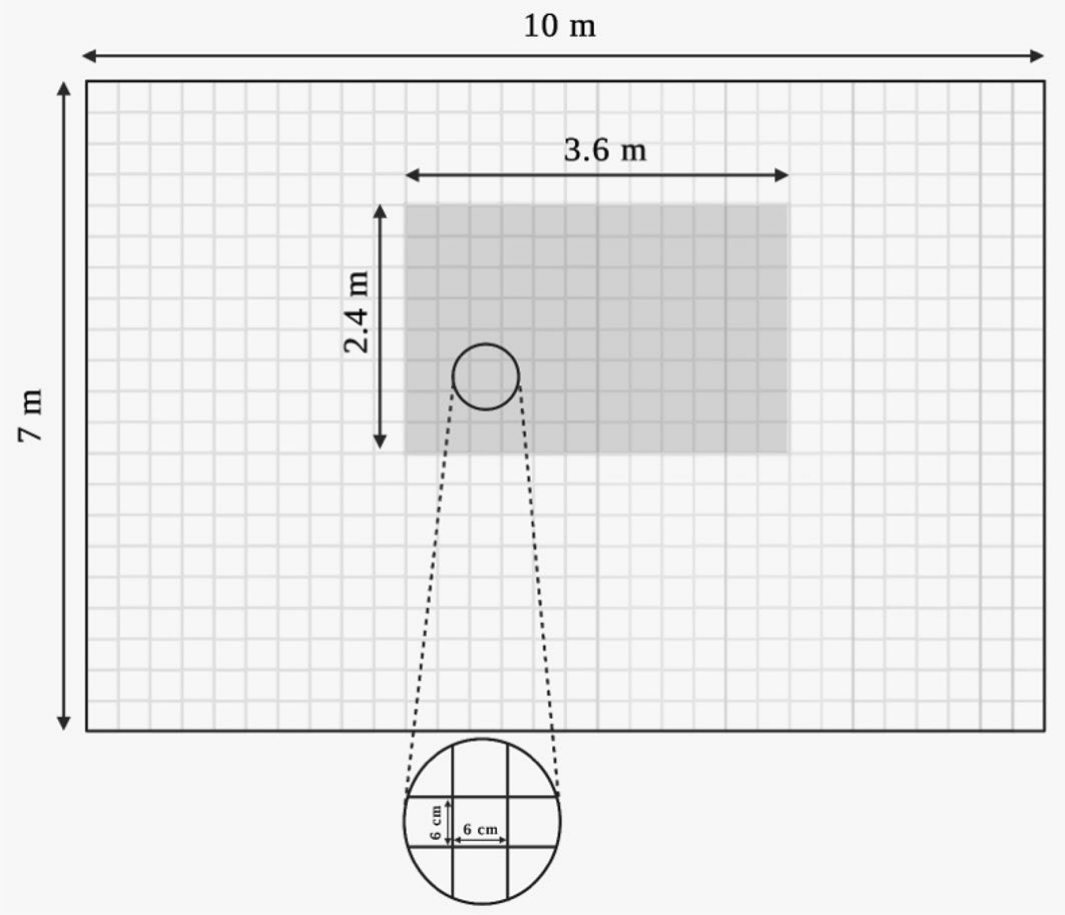
The computational domain and cell size (not to scale, for illustration purposes only), the cell size was homogonous throughout the domain

**Domain and Cell Size:** The domain of each model was 5.0 m × 6.0 m × 4.0 m (X, Y, Z) accompanied with a cell size of 6 cm for the whole domain. Therefore, it was decided to choose the cell size based on the wood crib design and according to the method proposed by Kallada Janardhan and Hostikka [34]. A cell size check was however conducted using the method proposed by Ma and Quintiere [35] namely the D^*^ method where D^*^ is calculated using Eq. 1:

1 $$D^{*} = \left( {\frac{{\dot{Q}}}{{\rho_{\infty } T_{\infty } C_{p} \surd g}}} \right)^{{{\raise0.7ex\hbox{$2$} \!\mathord{\left/ {\vphantom {2 5}}\right.\kern-0pt} \!\lower0.7ex\hbox{$5$}}}}$$

where D^*^ is the characteristic length scale of the fire plume, $\dot{Q}$ is the HRR, ${\rho }_{\infty }$ is the ambient air density,${T}_{\infty }$ is the ambient air temperature, ${C}_{p}$ is the specific heat of air and $g$ is the gravitational acceleration. Based on the D^*^ the cell size to be used in the simulations, should be less than 0.1 D^*^. Assuming a HRR of 3000 kW (since the focus of this paper is the fully developed phase of the compartment fire, the lowest average HRR within the fully developed phase, 3000 kW, from the experiments of different scenarios was used to compute the D* correlation), the recommended cell size will be around 16 cm where the cell size used in these simulations (i.e., 6 cm) is relatively smaller. Although the D^*^ method was derived based on pool fires, it also showed good performance for numerical scenarios with wood cribs as reported by other researchers [36, 37]. This approach is similar to those found in literature on the modelling of full-scale ISDs [38]. Cicione and Walls [39] conducted a cell sensitivity study to model the fire spread between multiple dwellings with very close conditions to the ones in this study and it was found that a 0.1 m cell size was sufficient to capture the gas layer temperature, heat fluxes to the surroundings and the lining materials burning behaviour. The size of the domain and location of the compartment is illustrated in Fig. 7.

**Simple Pyrolysis Model:** The heat release rate per unit area (HRRPUA) of the wood (pine wood) was taken from specific cone calorimeter studies [10] on ISs materials and assuming a heat flux of 75 kW/m^2^. The ignition temperature of the wood was set to be 250 °C [40]. The heat of combustion was taken as 20 MJ/kg [41] for the dominating fuel (pine wood). The chemical composition of the wood was set as input for the FDS as C_3.4_H_6.2_O_2.5_ [42] with a soot yield of 0.015 [43]. The accelerant/igniters (gasoline’s) HRRPUA curve used in the tests, was based on the early stage HRR curve of the tests in Wang et al. [14]. A sensitivity analysis for these inputs has been discussed by Cicione and Walls [39].

**Materials Thermal and Physical Properties:** The wood’s bulk density was chosen as 535 kg/m^3^ [41], and was adapted to 455 kg/m^3^ based on the cell size according to the method proposed by Kallada Janardhan and Hostikka [34], while the specific heat was assumed to be 1.3 J/Kg, and the conductivity to be 0.2 W/m.K. The steel was assumed to have a specific heat of 0.6 J/Kg, conductivity of 45 W/m.K, density of 7850 kg/m^3^, and emissivity of 0.23 (Steel Galvanised New) [44].

**1-D Conduction Heat Transfer:** The steel sheets were of thickness of 0.5 mm which is also the thickness of the 1-D heat transfer model. Since the steel walls thickness (0.5 mm) was much less than the cell size, therefore, the walls back side condition was set as EXPOSED—FDS command—for the 1-D heat transfer interaction between the two sides of the wall.

**The Instrumentation: Incident Radiative Heat Flux:** The incident radiative heat flux was measured in the experiments using the Thin Skin Calorimeter (TSC) which was developed with an intend to provide practical, low-cost device enabling the cost-effective mass production required for characterising the thermal boundary conditions during multiple large-scale fire tests. The technical description of the TSC design and a formulation of the proposed calibration technique are presented in Hidalgo et al. [33]. The incident radiative heat flux in the model was modelled using the ‘RADIATIVE HEAT FLUX’ output quantality which is related to the thermal exposure of solid surfaces which calculates the net radiative component of the following equation: ${\dot{q}}_{r}^{{\prime}{\prime}}={\mathcal{E}}_{s}({\dot{q}}_{inc}^{{\prime}{\prime}}-\sigma {T}_{s}^{4})$. Where ${\mathcal{E}}_{s}$ is the emissivity of the surface, ${\dot{q}}_{inc}^{{\prime}{\prime}}$ is the incident heat flux, $\sigma$ is Stefan-Boltzmann constant and ${T}_{s}$ is the surface temperature. **Gas temperature (Thermocouples):** The output quantity ‘THERMOCOUPLE’ is used to model the thermocouples. The thermocouple temperature lags the true gas temperature by an amount determined mainly by its bead size. The same bead diameter (i.e., 1.0 mm) for the experiments were used in the model, the default emissivity of 0.85 and the default values for the bead density and specific heat of nickel; 8908 kg/m^3^ and 0.44 kJ/kg/k, respectively was used in the model. **Gas Velocity (v-velocity):** The gas velocity component $v$ was modelled using the point device (DEVC). The $v$ component represents the velocity of gas flow normal to the plane of the compartment door. This approach mimics the physical placement and operation of the flow probes used in the experiments. **Gas Concentration (O**_**2**_**):** In the experiments, oxygen concentration was measured by extracting gas samples using probes placed at the previously mentioned locations (more information regarding the Oxygen cell in comment 3). In the numerical model, we replicated this setup by defining sampling point that calculates the local oxygen concentration from the simulated species transport equation at those locations. Velocity and temperature Slices were set vertically in the middle of the openings (windows and door) for each scenario.

**Leakage:** To model leakage through the compartment’s boundaries, FDS uses two alternative methods; the first is based on using pressure zones, while the second uses HVAC vents. In this paper, leakage is referring to any air that escapes through small gaps (i.e., the gaps between the walls and wall with the roof). In most cases, the leakage area is smaller than the cell size, therefore, the gaps are not modelled directly. In these cases, the HVAC model was used to connect the leaking compartment to the surroundings. In compartment fires, it is assumed that the most influential corrugation flutes are those at the top of the compartment, as hot gases will escape through these flutes due to the buoyancy effect, effecting both the time to, and Heat Release Rate (HRR) needed for, flashover. Additionally, it was observed in the experiments that most of the leaking external flames were at the top of the walls. Therefore, the leakage was only modelled at the top of the walls.

## Results and Discussions

The results and discussions will be presented by group. In the analysis of the experimental data, flashover, has been defined as the sudden propagation of flames out of the compartment. The external plumes were tracked using recorded videos for each experiment and the exact time for flashover was recorded based on this criterion. The numerical flashover criterion is assumed to be the time when the heat flux measurements on the four corners of the compartment’s floor reaches 20 kW/m^2^ [45]. The different criteria are used for two reasons; first, no heat flux measurements were made on the floors of the experiments conducted (given the complexity and potential error to measure such parameter in a compartment fire with wood crib); and second, in a pervious study by the authors (Beshir [46]), it was found that the top gas layer temperatures observed in the experiments when external flaming was observed were in some cases below the commonly used 525 °C hot gas layer temperature, therefore it was not possible for these types of compartments to rely on the well-known 500–600 °C hot gas layer temperature as a criteria for flashover [45]. For these thermally thin bounded compartments, flashover is driven not only by the hot gas layer, but also the reradiation from the hot walls. Beshir [46] provides a verification that using these two different criteria (external flaming for experiments and 20 kW/m^2^ on the compartment floor) provides accurate time to and ${\dot{q}}_{fo}$ for flashover.

### Group 1: Location of Window Relative to Door

#### Group 1: Experiments

**Fig. 8:**
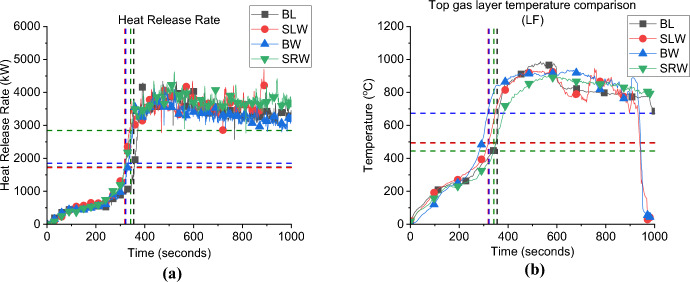
**a** the Heat release rate and **b** gas layer temperature at the left front top thermocouples (dashed lines highlights the flashover time, temperature and HRR), *note*: MB top TC is used for the BL case as most other TC trees were corrupted

In this study, it is postulated that two ventilation aspects can play a role in the fire dynamics: the window location (during the fuel-controlled phase) and the flow type (perpendicular or crossflow, during the ventilation-controlled phase). Numerical simulations are used to inform any gaps of knowledge in relation to these cases. The HRRs are compared for the cases; side left wall—SLW, back wall—BW, and side right wall—SRW, with the BL as demonstrated in Fig. 8a.

**Growth Phase:** the BL showed the lowest ${\dot{q}}_{fo}$ (HRR needed for flashover to occur) value to reach flashover (1.5 MW), while the SLW and BW required 1.7 MW and 1.9 MW, respectively. However, due to the delayed ignition of crib 2 nearest the door (delayed by around 60 s from the ignition of crib 1 due to a technical issue) the SRW case reached flashover at HRR of 2.9 MW. The BL case reached flashover at 355 s while the SLW, BW and SRW needed around 319, 332 and 342 s, respectively. Results for SRW should be considered with caution, especially for the time and HRR needed for flashover. Further investigation is needed to understand why there is such a discrepancy between the HRR to reach flashover for the SRW and other cases in this group, particularly understanding the uneven fuel burning within the compartment and how this interacts with the openings, leakage, air flow and the heat losses through the thermally thin walls.

This indicates that locating the window opening in a wall other than the front wall (where the door is located) leads to slightly faster time to flashover and considerably higher ${\dot{q}}_{fo}$. At the same time, while the ${\dot{q}}_{fo}$ of SLW and BW cases are higher than the BL case with longer time to reach flashover, the differences between the two cases in terms of the ${\dot{q}}_{fo}$ or the time to flashover are insignificant.

For the post-flashover phase: when the compartment is ventilation controlled, the flow type between the window and the door (i.e., perpendicular, or crossflow) will play an important role on the peak HRR that the compartment can reach. The average HRR for the SLW and SRW (perpendicular flow) was around 3.8 MW, whereas the BW case (crossflow) presented a slightly lower averaged HRR of around 3.4 MW. One of the main reasons is believed to be the crossflows that resist each other instead of improving the mixing process, resulting in lower burning efficiency post-flashover. While the higher peaks for the SLW and SRW cases can be due to a higher time scale for flow mixing, and results in better turbulent mixing resulting in higher HRR peaks. Figure 8b depicts the gas layer temperatures measured at the top left front corner TC tree of each case (i.e., TC LF_10). The results show that the maximum temperature of all cases was around 900 °C, even with the different window locations, the top hot gas layer seems to be homogeneous in all cases.

**Fig. 9:**
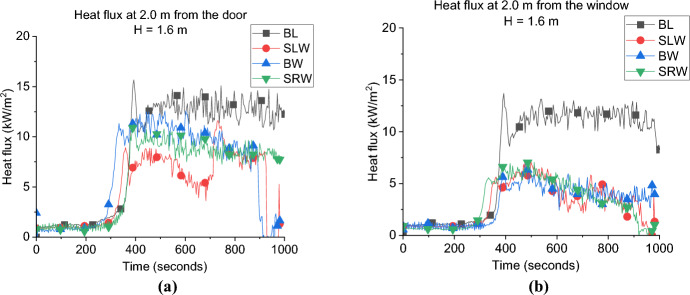
The external radiative heat fluxes of Group 1: **a** at 2.0 m from the door at 1.6 m height and **b** at 2.0 m from the window at 1.6 m

**Door Heat Flux:** The incident radiative heat flux measurement of each case at 2.0 m from the door and 1.6 m height is presented in Fig. 9a. It was found that having the window on a wall other than the wall with the door significantly reduced the measured radiative heat fluxes opposite the door. Where the average values for the heat flux from the door post-flashover were found as 7.3, 8.8 and 10.1, kW/m^2^ for the SLW, SRW and BW, respectively, compared to around 12.8 kW/m^2^ for the BL. This was, however, expected as the heat flux measurements opposite the door in the BL case also captures the heat flux from the external plume out of the window.

**Fig. 10:**
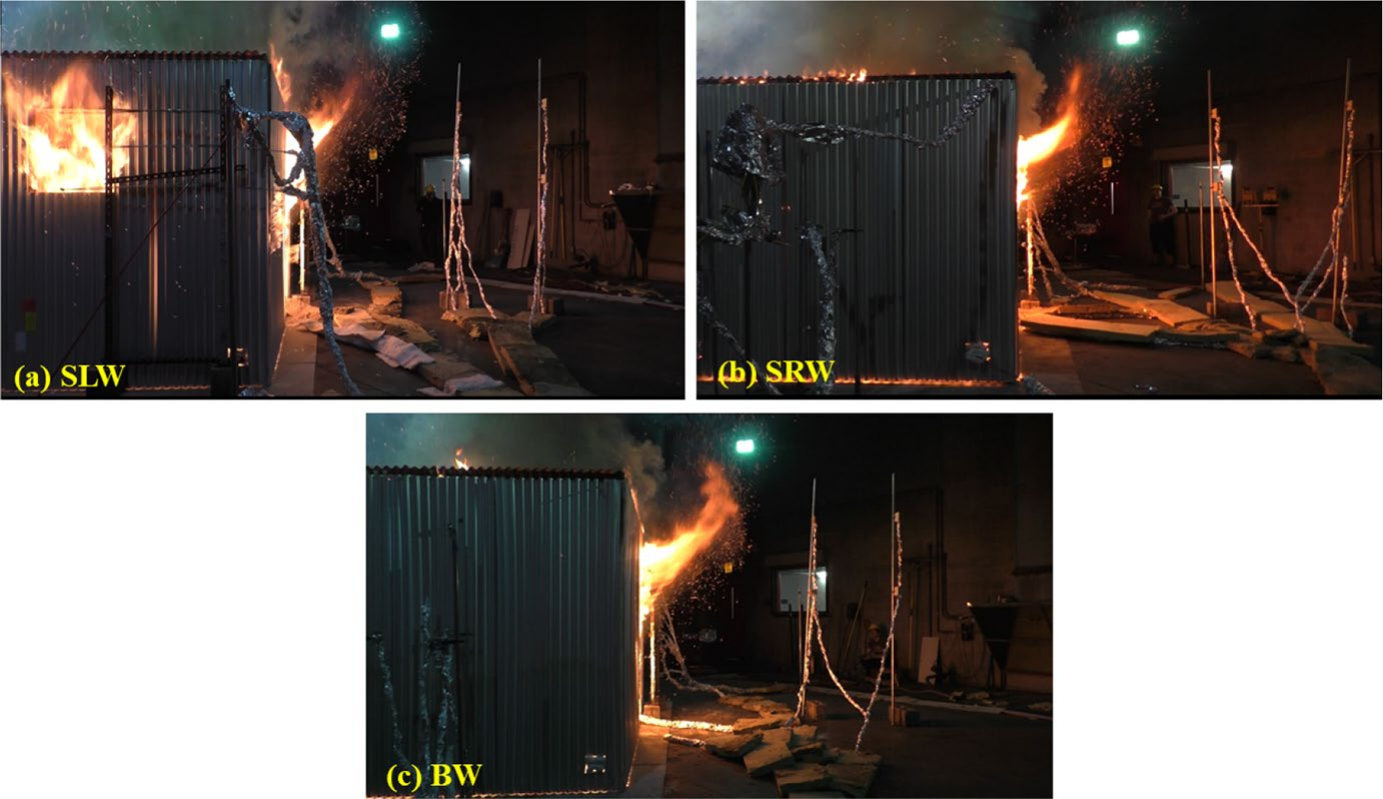
Door external plume from the door post-flashover for: **a** SLW, **b** SRW and **c** BW cases

Comparing the three cases SLW, SRW and BW, it shows that placing the window opposite to the door gives the highest heat flux opposite the door. The crossflow was found to affect the mixing/air circulation and hence the combustion efficiency is expected to decrease within the compartment and led to lower peak HRR. The less the mixing within the compartment led to more unburnt gases leaving through the door and as shown in Fig. 10 the BW case experienced the longest external plume from the door in the three cases. The contrary is observed for SLW case, where the inlet air through the door circulates much better due to the perpendicular flow (with window far from the door), led to highest peak HRR and shortest external plume from the door (less unburnt gases).

**Window Heat Flux:** The incident radiative heat fluxes at 2.0 m from the window and 1.6 m height are presented in Fig. 9b. The results illustrate that locating the window in a wall other than the front wall (where the door is located) will decrease the maximum heat flux to almost half of its value. As it is presented the average heat fluxes are around 4.0 kW/m^2^ for cases SLW, SRW and BW, compared to around 12.2 kW/m^2^ for the BL case. This again shows the effect of adding two openings on the same wall, the external plume from the door interacting with the heat flux measurements from the window.

The data also shows that placing the window anywhere other than the front wall does not change the peak, or average, heat flux from the window.

**Fig. 11:**
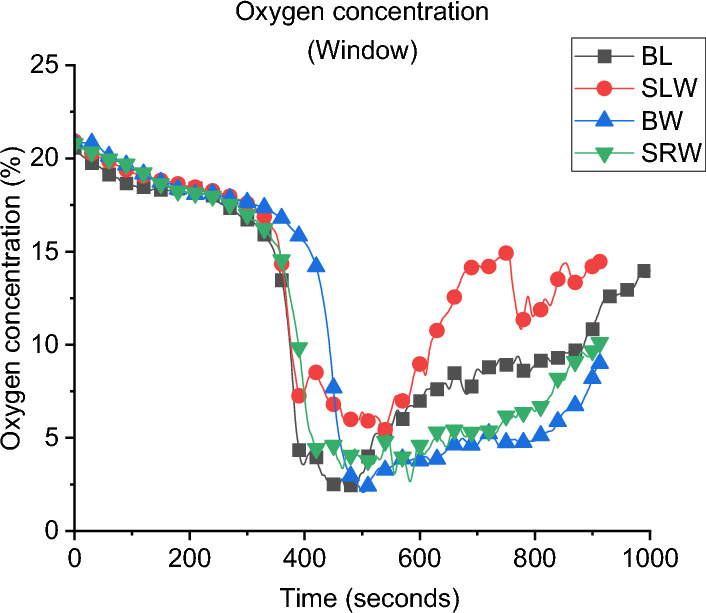
Oxygen concentration at the window for all cases of Group 1

**Gas Concentration:** The oxygen gas concentrations for all cases are presented in Fig. 11. During the post-flashover phase, the BW case has the lowest oxygen concentration near the window which is evident of the higher burning rate at the external plume due to the crossflow. While the SLW showed the highest oxygen concentration which indicates lower amounts of unburnt gases leaving the compartment and hence less burning rate at the window.

#### Group 1: Numerical Modelling

**Fig. 12:**
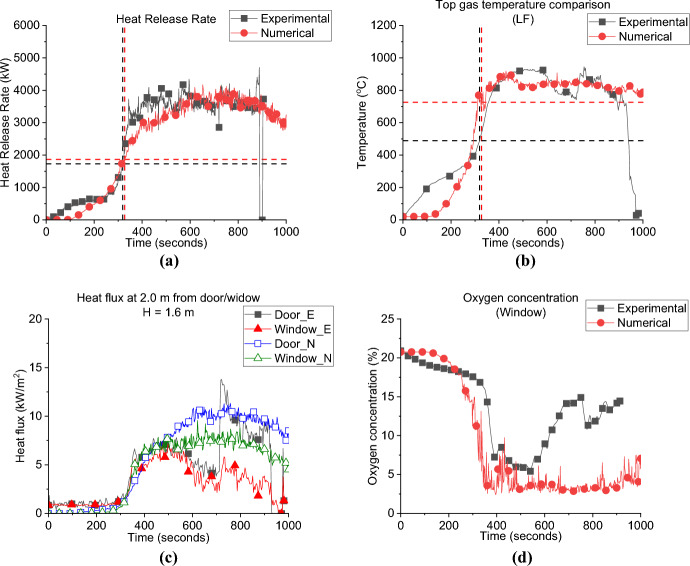
Model vs. experimental results for SLW case: (**a**) Heat Release Rate, (**b**) Left Front top thermocouple, (**c**) Heat flux at 2.0 m from door and window at height of 1.6 m and (**d**) Oxygen concentration at the window of the compartment (dashed lines marks the flashover time, temperature and HRR for each case, black for experimental and red for numerical)

Figure 12 presents the comparison between the numerical and experimental results for the SLW case. The FDS model captures the same HRR curve (e.g. trend) as presented in Fig. 12a, and, as presented in Table 2, well both the ${\dot{q}}_{fo}$ and time to flashover values of 1.9 MW and 327 s, respectively. The gas layer temperature was also well captured at a variation of around ± 5%, as presented in Fig. 12b. The FDS results for the heat fluxes from the door and window, simulated at 2.0 m and 1.6 m high, correlate well with the experimental results from flashover up to 500 s. However, from around 600 s, onwards the FDS model overestimated these fluxes, as presented in Fig. 12c. At that time (600 s and beyond) post-flashover, the thin steel sheet walls were starting to warp, allowing greater air entrainment and areas of ejected flames from the new generated gaps, thus reducing the amount of unburned gases leaving the compartment from the window/door and hence the measured radiative heat fluxes at the openings during the experiment. This changing ventilation was not captured in the model. Figure 12d presents the oxygen concentration at the window experimentally and numerically, FDS overestimated the oxygen consumption at the window compared to the experimental results, which again could be related to the sheet walls starting to wrap leading to move fresh air entering the compartment and increasing the oxygen concentration during the experiments.

**Table 2:** Comparison between experimental and numerical results for Group 1: neutral plane, HRR at flashover and time to flashover

Case	Neutral plane (E/N) [m]	HRR at flashover (E/N) [MW]	Time to flashover (E/N) [seconds]
BL	0.95/0.95	1.5/2.0	355/334
SLW	1.05/1.05	1.7/1.9	319/327
BW	−/0.90	1.9/2.0	322/333
SRW	−/1.05	2.9/2.0	342/350

**Fig. 13:**
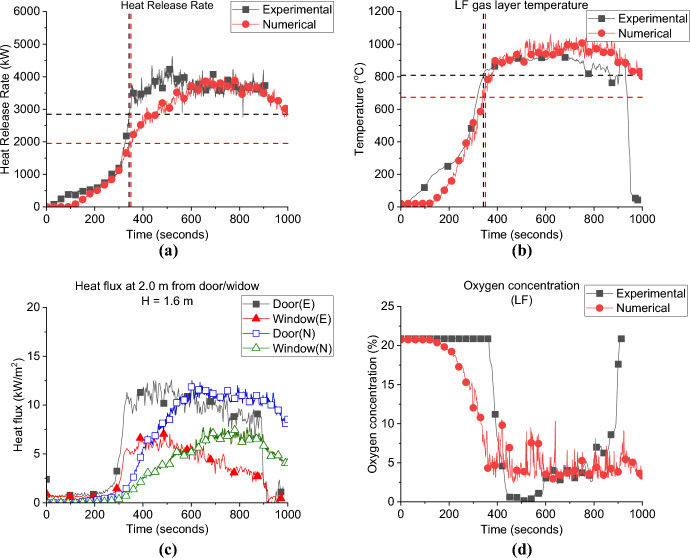
Model vs experimental results for SRW case: (**a**) Heat Release Rate, (**b**) Left Front top thermocouple, (**c**) Heat flux at 2.0 m from door and window at height of 1.6 m and (**d**) Oxygen concentration at the front of the compartment (dashed lines marks the flashover time, temperature and HRR for each case, black for experimental and red for numerical)

**Fig. 14:**
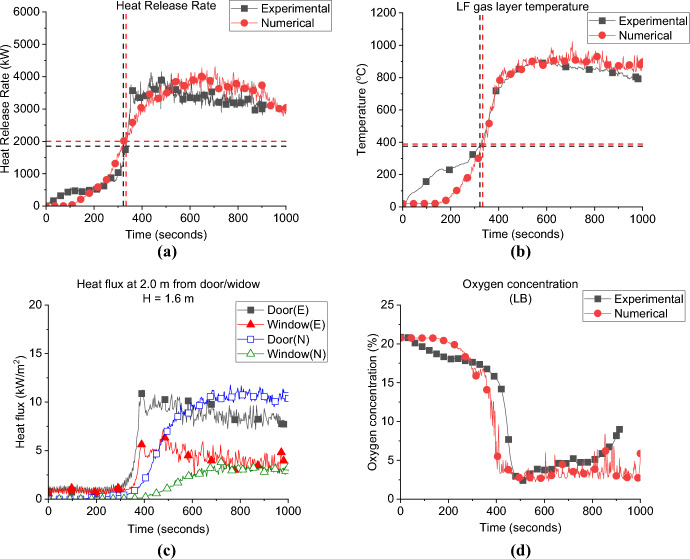
Model vs experimental results for BW case: **a** Heat Release Rate, **b** Left Front top thermocouple, **c** Heat flux at 2.0 m from door and window at height of 1.6 m and **d** Oxygen concentration at the back of the compartment (dashed lines marks the flashover time, temperature and HRR for each case, black for experimental and red for numerical)

Figure 13 presents the same data as Fig. 12, but for the SRW experiment. Figure 13a shows the HRR-time curve was again well replicated by the FDS model and time to flashover were estimated accurately compared to the experimental results as 350 s (i.e., 2.5% variation), as presented in Table 2. Due to the technical issue, ${\dot{q}}_{fo}$ from the experiment was found to un-realistically high (i.e., 2.9 MW). It was also estimated numerically that the ${\dot{q}}_{fo}$ of this case is around 2.0 MW, comparable to the SLW case’s value. The model also managed to simulate the gas layer temperature as shown in Fig. 13b. The peak heat flux value at the door was well predicted by FDS with a delay in time, while the window peak was underestimated with the same increment of delay as presented in Fig. 13c. Figure 13d presents the oxygen concentration at top front of the compartment, where it was found that FDS slightly overestimates the oxygen concentration. Most of the aspects in the experimental data were well replicated by FDS for the BW case as presented in Fig. 14. Figure 14a and Table 2 shows that the HRR-time curve, the time to flashover and the ${\dot{q}}_{fo}$ matches well with the experimental data, with variation of only 1% and 8% for the time to flashover and ${\dot{q}}_{fo}$, respectively. Figure 14b shows that the averaged fully developed phase heat flux at the door and window matches well with the numerical results, however, the same delay observed in the SRW case is observed in BW. NB: due to a malfunction in the flow probes—no neutral plane height was measured for the SRW and BW experiments.

The differences between the BW (i.e., crossflow) case and the SLW (i.e., perpendicular flow) cases, found experimentally are confirmed numerically, namely, the heat fluxes measured at the door of the BW case was also found to be significantly higher than that of the SLW case.

**Fig. 15:**
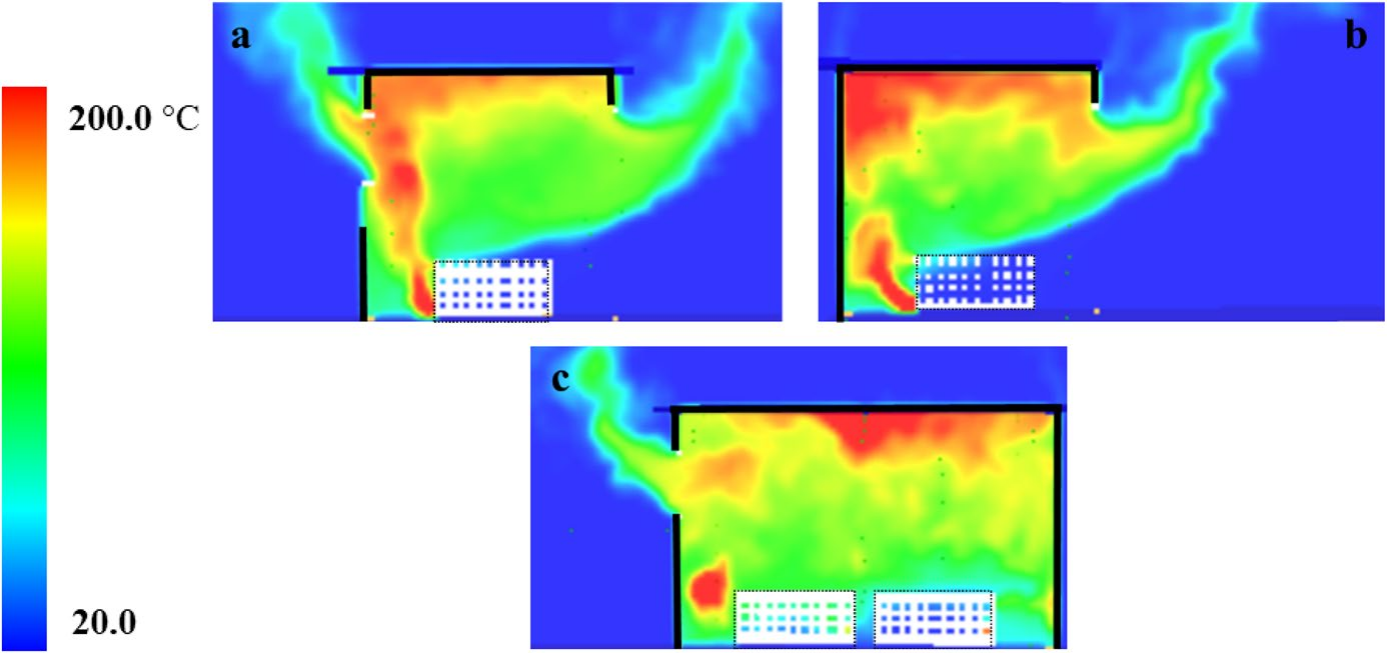
0–200 s (pre-flashover) averaged temperature slice files for Group 1: **a** perpendicular on the front wall—on the window’s centre line- for BW case, **b** perpendicular on the front wall—on the door’s centre line—for SLW and **c** perpendicular on the left wall—on the window’s centre line- for SLW case

When comparing the temperature slice files in Fig. 15a/b, it can be observed that the presence of the window on the back wall led to more flow to leave the compartment via the door as seen in Fig. 15a/b, while heat was accumulated on the back wall for the SLW case. Figure 15c, shows the heat flow leaving the side window in case SLW, which is less than or equal to that observed for the BW case window. However, the overall external flows were more for the BW given the higher flow from the door. Therefore, it can be concluded that crossflow leads to more flow/heat losses through the vertical openings pre-flashover and that led to slightly more heat required for this compartment to reach flashover, observed in the experiments.

**Fig. 16:**
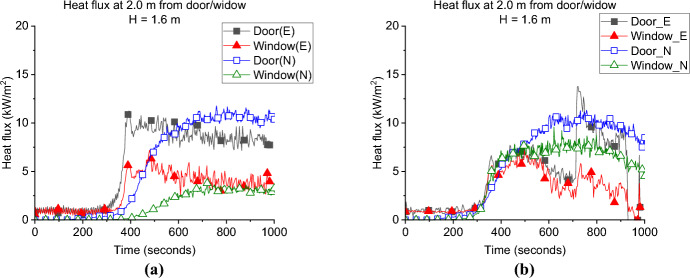
Measured and modelled heat flux at 2 m opposite the door and window (height 1.6 m) for Group 1: **a** BW case, and **b** SLW case

**Fig. 17:**
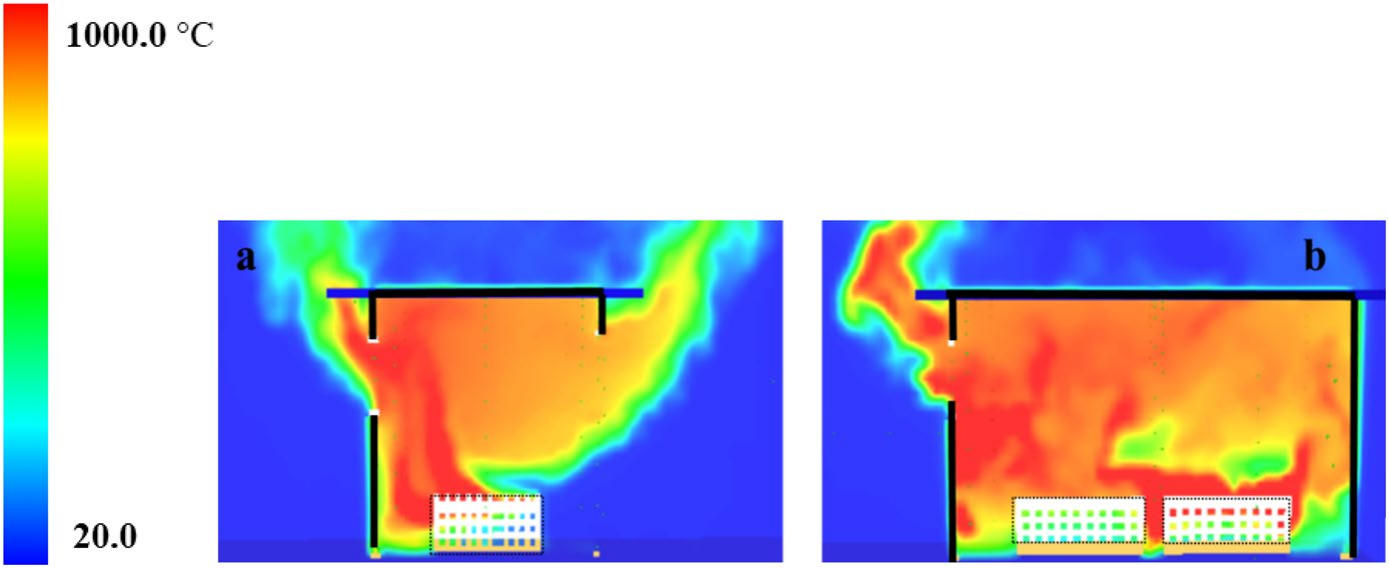
400–500 s (post-flashover) averaged temperature slice file for Group 1: **a** perpendicular on the front wall—on the window’s centre line—for BW case, **b** perpendicular on the left wall—on the window’s centre line—for SLW case

For external recorded heat fluxessimulated in front of the window (see Fig. 16) in both cases, post-flashover, Fig. 17 shows a comparison of the averaged external plume size from the window for the BW and SLW cases (plume averaged for 100 s from 400 to 500 s for both cases). It is shown that the external plume size is almost the same for both cases and same for the heat fluxes simulated at the window of each case. Therefore, placing the window on the back wall will lead to shorter neutral plane as presented in Table 2 and hence bigger plume from the door, higher heat fluxes, and more heat required to reach flashover. However, it will not have significant effect on the time to reach flashover, heat fluxes from the window, and the size of the window’s external plumes.

### Group 2: Double Window

#### Group 2 Experiments

In this section, the effect of adding an extra window to the left wall of the BL case (DW) was investigated. The results from the DW case were compared to mainly BL case with some aid from the SLW case at some points.

**Fig. 18:**
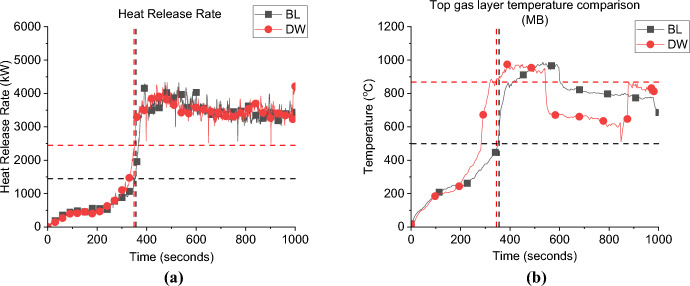
**a** Heat release rate and **b** Gas layer temperature at the middle back top thermocouples (dashed lines marks the flashover time, temperature and HRR for each case)

**Growth Phase:** Fig. 18a presents the HRR curve of the DW case compared to the BL. It was found that the ${\dot{q}}_{fo}$ of DW case (i.e., 2.2 MW) is much higher that the BL case (i.e., 1.5 MW). However, the time to reach flashover is roughly the same for both cases, around 350 s. Clearly, the influence of increasing the ventilation factor, significantly increased the heat losses from the compartment in the growth phase and more HRR was needed for the same compartment to reach flashover. This difference in ${\dot{q}}_{fo}$ was also spotted in the comparison between the BF and NW cases from Beshir et al. [32] which compared ${\dot{q}}_{fo}$ between two cases with fuel at the back of the compartment, with the same set-up as here, but with the NW case not having a window (note: BF was same as BL but with fuel at the back of the compartment). The NW case required a significantly lower ${\dot{q}}_{fo}$ and had a slightly longer time to flashover. Therefore, from both studies, it can be concluded that in these compartments (as expected), the ventilation factor is directly proportional to the ${\dot{q}}_{fo}$ and is no different than the thermally thick non-leaky compartments.

**Post-flashover:** It was also found that with higher ventilation factor the effect of the perpendicular flow (for the second window) is negligible and as it was found that both the average HRR and top gas layer temperatures matches for BL and DW, with no observed effect for the extra ventilation.

**Fig. 19:**
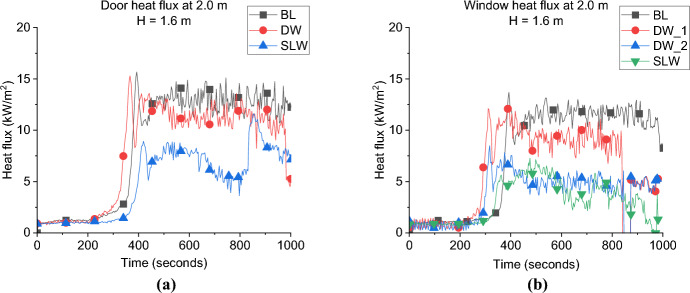
The external radiative heat fluxes of Group 2: **a** at 2.0 m from the door at 1.6 m height and **b** at 2.0 m from the window at 1.6 m (*note*: DW_1 is at the front wall)

**Door Heat Flux:** The incident heat flux measurement for each case at 2.0 m from the door at 1.6 m high, presented in Fig. 19a, show that the averaged incident heat flux of DW case is slightly lower (i.e., 12.0 kW/m^2^) than the BL case (i.e., 14.0 kW/m^2^), therefore, it can be demonstrated that increasing the ventilation factor has slight effect on the heat flux from the door. The same was found in the comparison between the BF and NW cases from Beshir et al. [32].

The SLW case still shows much lower values compared to the DW case, which proves that one of the main reasons (in addition to the flow field) for the high differences in the heat fluxes from the opening of the BL and the other cases in Group 1 was due to the dual effect from the window and door on the heat flux measured in front of each opening when placing both openings on the same wall.

**Window Heat Flux:** The incident heat flux measurement for each case at 2.0 m from the window at 1.6 m high is presented in Fig. 19b, and show that increasing the ventilation factor will slightly reduce the average incident heat flux of around 12 kW/m^2^ from the front wall window for the DW case, compared to around 13 kW/m^2^ for the BL case. It was also found that both cases reached the peak heat fluxes at the nearly the same time post-flashover. For the side window, it was found that the average value reached by the DW was slightly lower to the SLW case.

**Fig. 20:**
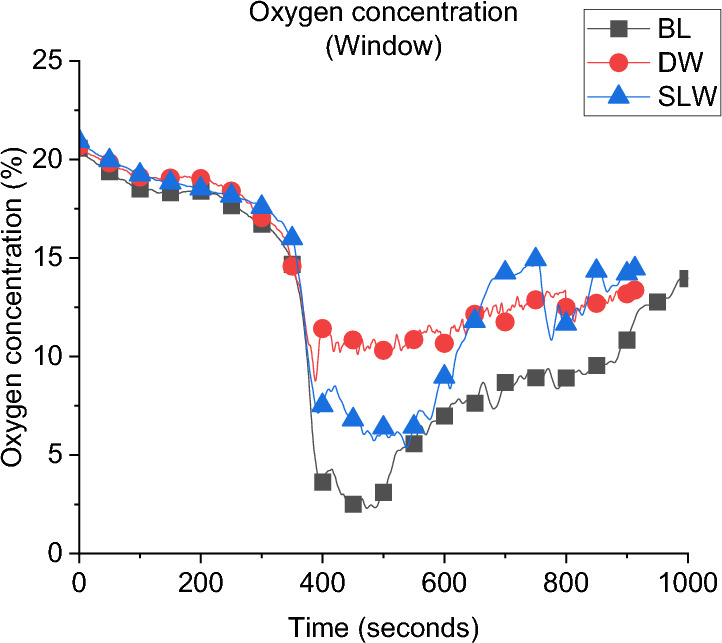
Oxygen concentration at the window for Group 2

**Gas Concentration:** The concentration of oxygen at the window of DW, SLW and BL cases is presented in Fig. 20. It was found that increasing the ventilation factor (i.e., DW case) led to higher oxygen concentrations at the left wall window. The increased availability of fresh air (i.e., oxygen), combined with the enhanced mixing within the compartment due to the added ventilation, led to more efficient burning rate within the compartment. This resulted in reducing the amount of unburnt gases leaving and increasing the oxygen concentration at the DW case. This is also linked to the slightly lower heat fluxes (e.g., external plume intensity) observed from the left window in the DW case compared to the SLW case.

#### Group 2: Numerical Modelling

**Fig. 21:**
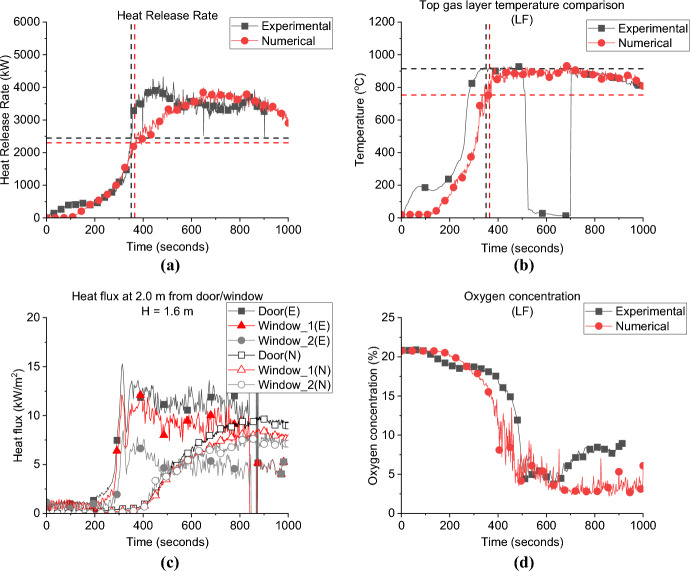
Model vs experimental results for DW case: **a** Heat Release Rate, **b** Left Front top thermocouple, **c** Heat flux at 2.0 m from door and window at height of 1.6 m and **d** Oxygen concentration at the front of the compartment (dashed lines marks the flashover time, temperature and HRR for each case, black for experimental and red for numerical)

Figure 21a presents the HRR-time curve for the DW case, numerically and experimentally. It was found that the HRR average values were well-estimated by the model. Additionally, Table 3 shows that the time and ${\dot{q}}_{fo}$ were well estimated by the model with approximately ± 5% variations. The averaged steady state top gas layer temperature at the LF top thermocouple was also well replicated by the model, as presented in Fig. 21b. The external heat fluxes were again delayed compared to the experimental results with the door and front wall window peak values well replicated, however, the left wall window (window 2) values were clearly overestimated, as presented in Fig. 21c. The oxygen concentration at the top front of the dwelling was well captured by the model post-flashover as presented in Fig. 21d.

**Table 3:** Comparison between experimental and numerical results for Group 2: neutral plane, HRR at flashover and time to flashover

Case	Neutral plane (E/N) [m]	HRR at flashover (E/N) [MW]	Time to flashover (E/N) [seconds]
BL	0.95/0.95	1.5/2.0	355/334
SLW	1.05/1.05	1.7/1.9	319/327
DW	−/1.175	2.5/2.3	350/365

### Group 3: Increased Ventilation Area and Aspect Ratio

#### Group 3: Experiments

In this section, the BL design was replicated but with different window designs. The first design was based on a vertical window (VW) of dimensions 1.2 m × 0.6 m, H × W and the second design is based on a horizontal window (HW) of dimensions 0.6 m × 1.2 m, H × W. The BL case had a 0.6 m × 0.6 m, H × W, window.

**Fig. 22:**
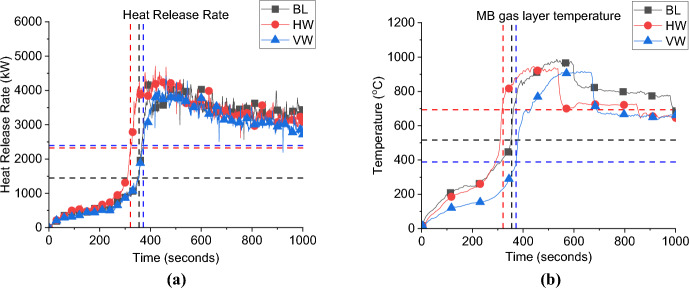
**a** Heat release rate and **b** Gas layer temperature at the left front top thermocouples (dashed lines marks the flashover time, temperature and HRR for each case)

**Pre-flashover:** Both cases are of ventilation factors ($${V}_{f})$$ higher than that of the BL case, therefore, the $${\dot{q}}_{fo}$$ for both cases is VW = 2.4 MW ($${V}_{f}=3.05)$$ and HW = 2.3 MW ($${V}_{f}=2.88)$$ (≈5% variation), compared to 1.5 MW for the BL case, as shown in Fig. 22a. This confirms what was found earlier, that increasing the ventilation factor directly impacts the value of the $${\dot{q}}_{fo}$$.

The aspect ratio, apparently, did not highly affected the ${\dot{q}}_{fo}$, however, it had a significant effect on the time to flashover. A taller narrower window (VW) led to 370 s to flashover, while the shorter wider window had a faster time to flashover of 320 s (≈ 15% variation) as shown in Fig. 22a. For the three cases, after around 200–250 s from flashover, the connections between the walls’ wood frames and steel sheets starts to loosen causing high leakage rate and hence sudden drop of temperatures as presented in Fig. 22b.

**Fig. 23:**
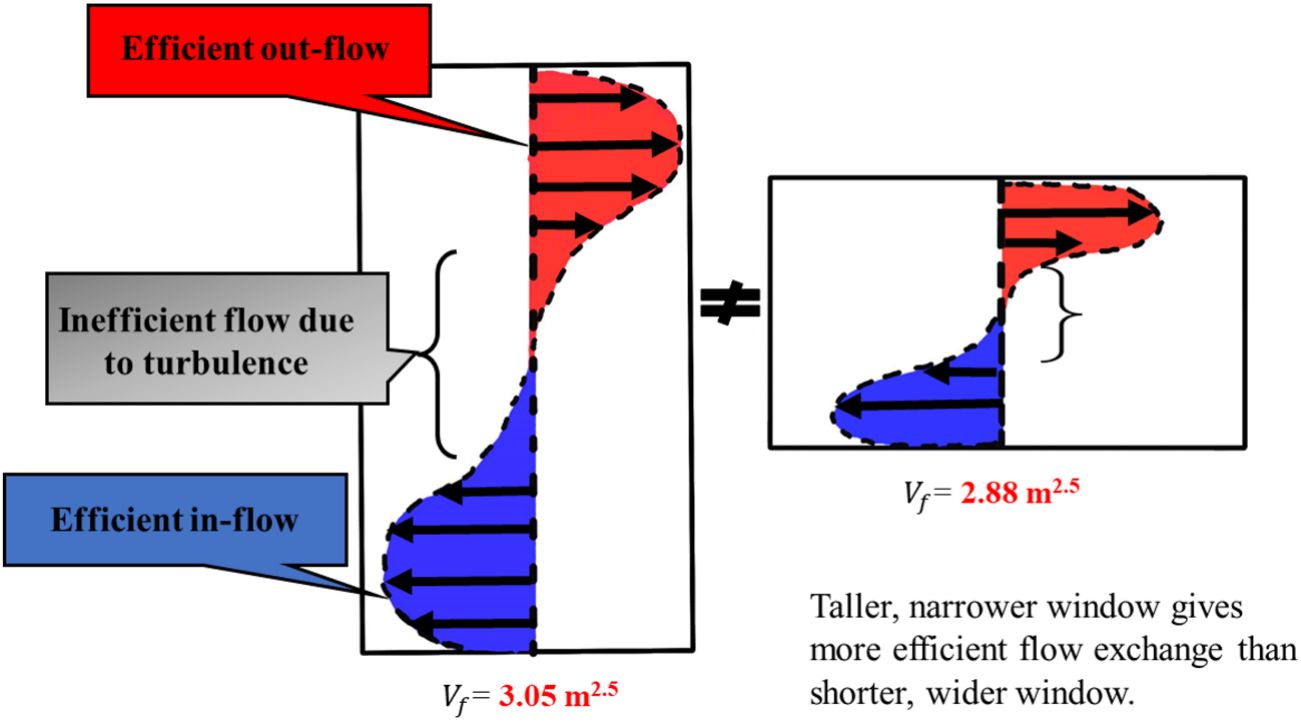
Flow field through vertical vs. horizontal opening (not to scale)

As explained in Fig. 23, taller, narrower window gives more efficient flow exchange than shorter, wider windows. The mixing and air entrainment play an important role on the time to flashover, therefore, the better the mixing, the more the air entrainment within the wood crib, which is expected to have a faster time to flashover. For that reason, the HW case, compared the VW case, had a faster time to flashover as there was more turbulent flow entering the compartment through the window and it is expected to enhance the mixing before flashover.

##### Post-flashover

Figure 22b represents the gas-layer temperature of the TC_10 of the MB TC tree for Group 3 cases against the BL scenario. It shows that BL case had slightly higher average top hot gas layer temperature, with no significant differences between the three cases, in terms of the peak or average values.

**Fig. 24:**
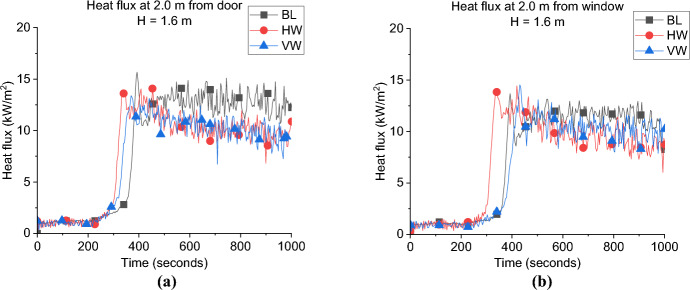
The external radiative heat fluxes of Group 3: **a** at 2.0 m from the door at 1.6 m height and **b** at 2.0 m from the window at 1.6 m

**Door Heat Flux:** The incident heat flux measurements at 2.0 m from the door and 1.6 m high of each case in Group 3 are presented in Fig. 24a and shows that increasing the ventilation factor significantly reduced the maximum incident heat flux at the door. However, the design of the window did not affect the heat flux at door, where the averaged heat fluxes from the door post-flashover are around 10 and 10.5 kW/m^2^ for the HW and VW cases, respectively compared to around 13.0 kW/m^2^ for the BL case.

**Window Heat Flux:** The incident heat flux measurements at 2.0 m from the window and 1.6 high is presented in Fig. 24b and shows that the average heat flux from the VW and HW cases was around 10.0 kW/m^2^, while the BL was slightly higher with 11.0 kW/m^2^. Therefore, it can be concluded that, the increase in the ventilation factor, slightly reduced the heat flux from the window and significantly reduced that for the door. However, the aspect ratio of the window (i.e., VW vs. HW) did not have almost any effect on the heat flux from the window or the door.

**Fig. 25:**
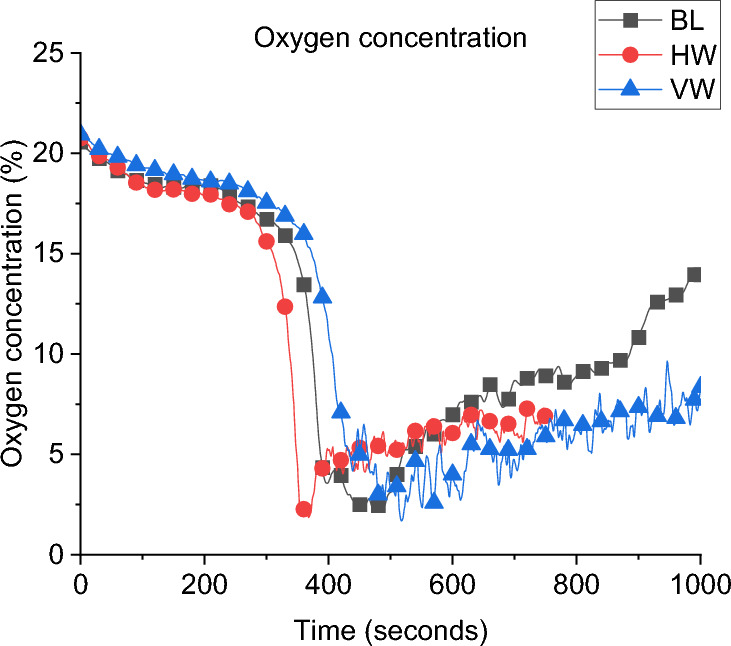
Oxygen concentration at the window for Group 3

**Gas Concentration:** As presented in Fig. 25 the oxygen concentration at the window of Group 3 compared to the BL case, the minimum oxygen concentration within the compartment is not overly affected by the orientation of the window, but it can be observed that after the minima, the BL case increased in O_2_ concentration at the measurement point, indicating more turbulent mixing from the smallest opening.

#### Group 3: Numerical Modelling

**Fig. 26:**
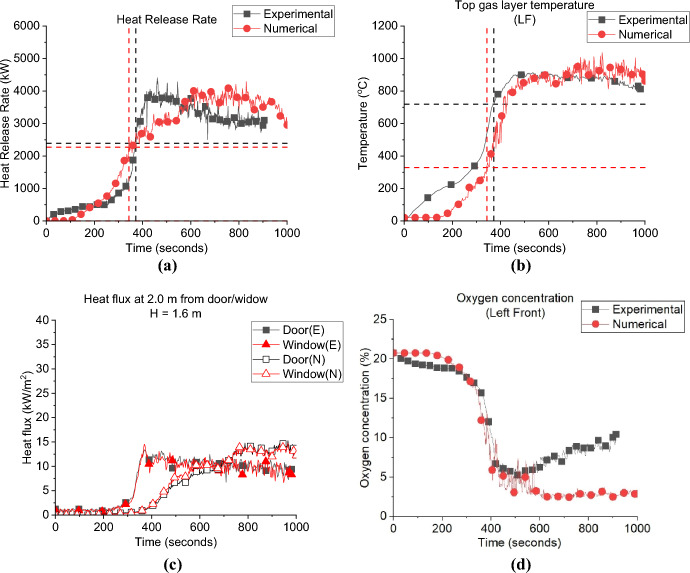
Model vs experimental results for VW case: **a** Heat Release Rate, **b** Left Front top thermocouple, **c** Heat flux at 2.0 m from door and window at height of 1.6 m and **d** Oxygen concentration at the front of the compartment (dashed lines marks the flashover time, temperature and HRR for each case, black for experimental and red for numerical)

**Fig. 27:**
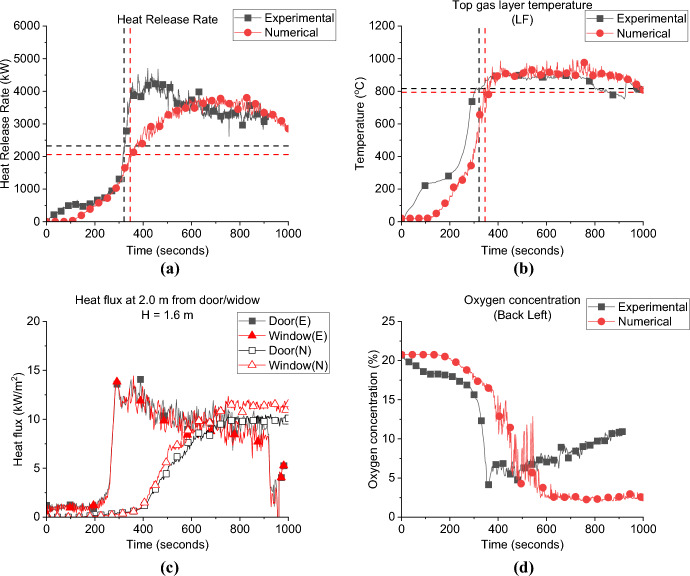
Model vs. experimental results for HW case: **a** Heat Release Rate, **b** Left Front top thermocouple, **c** Heat flux at 2.0 m from door and window at height of 1.6 m and **d** Oxygen concentration at the back of the compartment (dashed lines marks the flashover time, temperature and HRR for each case, black for experimental and red for numerical)

As presented in Fig. 26a and Table 4 the FDS model underestimated the time to flashover for the VW case by around 8.0%. However, as shown in Fig. 27a and Table 4 FDS slightly overestimated the time to flashover for the HW case. The averaged steady-state top gas layer temperature was well replicated at the top left front of the compartment for both cases as presented in Figs. 26b and 27b. The delay in the heat fluxes was observed again for both cases with the VW case’s averaged heat fluxes being slightly overestimated and those for the HW being well replicated as shown in Figs. 26c and 27c. The model very well replicated the trend of the oxygen concentration at the front of the compartment for the VW case, while it underestimated the oxygen concentration post-flashover at the back of the compartment for the HW case, as presented in Figs. 26d and 27d.

**Table 4:** Comparison between experimental and numerical results for Group 3: neutral plane, HRR at flashover and time to flashover

Case	Neutral plane (E/N) [m]	HRR at flashover (E/N) [kW]	Time to flashover (E/N) [seconds]
BL	0.95/0.95	1.5/2.0	355/334
VW	–/0.95	2.4/2.3	372/343
HW	–/1.175	2.3/2.1	321/346

**Fig. 28:**
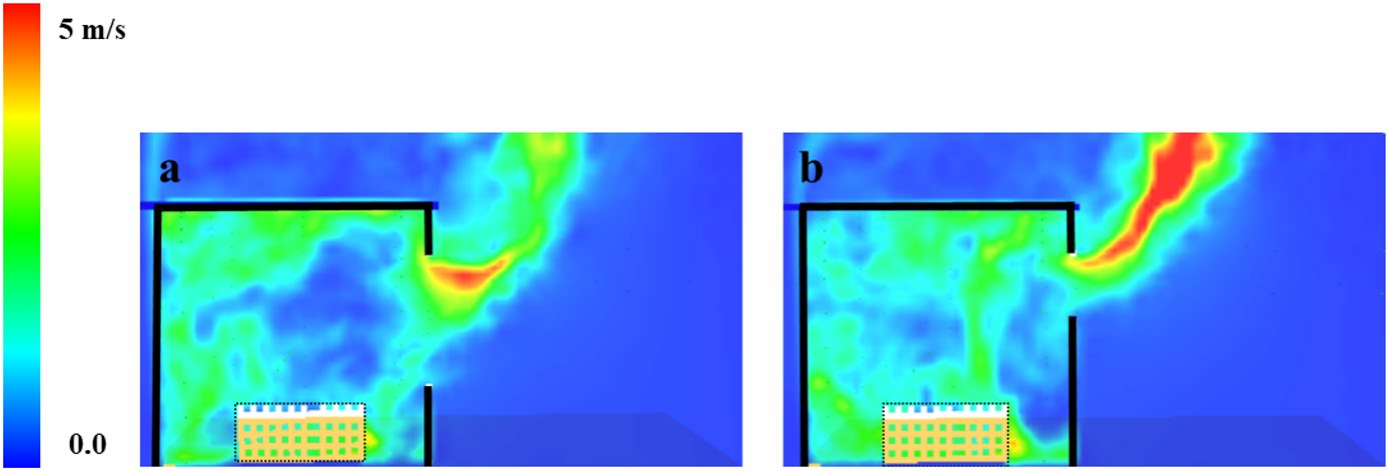
0–300 s (pre-flashover) averaged velocity slice files for Group 3 perpendicular on the front wall middle of the window for **a** VW case and **b** HW case

To check the flow field pre-flashover for both cases, the averaged velocity slice pre-flashover for each case is presented in Fig. 28. The velocities around the crib are much higher for the HW case compared to the VW case. The blue areas (which represents very low velocities) are more widespread for the VW case, meaning less entrainment and mixing compared to the HW case. These numerical observations confirm the reversion of the faster time to flashover of the HW case to the extra turbulent flow induced through the horizontal window, compared to the VW case.

## Summary and Discussion

The experimental and numerical modeling work presented above has provided a qualitative understanding of how the position and size of the window ventilation in relation to a door impacts the fire dynamics of these mock informal settlement dwellings. In this paper we investigated the effect of three key compartment-ventilation related parameters: (i) the ventilation factor (about 15% maximum difference), (ii) opening aspect ratios (for windows), and (iii) the positioning of ventilation openings. Our main aim was to understand their influence on the fire dynamics and the average heat fluxes expected from these compartment’s openings (doors/windows) post-flashover, particularly in the context of fire spread in informal settlements. This study revealed that the 15% variation in ventilation factor did not have a significant influence on both the average Heat Release Rate (HRR) or the heat fluxes from the doors and windows of each compartment. Where the observed differences in the two extremes (i.e., compartment with highest and lowest ventilation factor) ranged only between 1–2 kW/m^2^. Similarly, altering the aspect ratios of the windows, which resulted in average difference in heat fluxes within 2–3 kW/m^2^. However, a critical observation emerged regarding the placement of the window in relation to the door. Positioning the window on either side walls, as opposed to the front wall adjacent to the door, there was a relatively significant reduction in the heat flux measurements. Specifically, the heat flux measured at the door decreased by around 6 kW/m^2^ (approximated reduction of 45%), and at the window even higher by around 9 kW/m^2^ (approximated reduction of 70%). This was further investigated using the gas analysis measurements within the compartment and numerically using a CFD model and was attributed to altered fire dynamics within the compartment, driven by changes in the fresh air/external hot flow movement due to the window’s relocation. The numerical analysis indicated that this shift may have led to variations in mixing within the compartment, hence the distribution of the hot gases within the compartment and the size of the external plumes from windows/doors. These observations underscore the critical effect of the opening design (i.e., placement) in dictating the compartment fire dynamics and provide essential data needed for fire spread models in informal settlements.

It is also crucial to acknowledge the potential uncertainties associated with this work. As an example, identify the flashover time and HRR experimentally, we relied on observing when consistent external flames emerged from the compartment openings (i.e., door or windows). This is a classic straightforward method for comparative analysis; however, one main drawback of this method is the lack of error quantifications in literature. In our study, due to resource limitations, each experiment was conducted only once, hence restricting the ability to quantify the statistical relevancy of the accompanied uncertainties. As a layer of confidence to our experimental approach, we used the FDS model to replicate the experimental work. For example, the FDS model was used to determine the time/HRR onset flashover numerically for the same tests and there was a notable overlap between the two. Nonetheless, we still acknowledge the need for future research to develop a method to quantify the uncertainties in the fire dynamics predictions for large scale under-ventilated compartment fires, specifically those fuelled with solid fuels. Generally, this study directly aims to show the conclusive capabilities of the model in a qualitative way rather than a statistically based validation, defining prediction uncertainties of the model as recommended by the FDS validation guide [47] was not of the scope of this work. Based on that the conclusions/observations discussed in this study should be interpreted more for qualitative comparison purposes. However, recommended relative standard uncertainties mentioned in the validation guide are of high relevancy as they are related to the uncertainty in experimentally measured outputs and uncertainties in input parameters propagated to outputs, which both are part of our methodology. Accordingly mentioning the roots and the combined effect of the abovementioned uncertainties will give a good anticipation to the model’s capabilities as shown in Table 5.

**Table 5:** Relative standard uncertainties [47]

Measurement	Relative standard uncertainty of measured outputs (%)	Propagation of uncertainties from input to outputs (%)	Combined uncertainty (%)
Thermocouples	5	5	7
Gas analyzer	2	8	8
Bi-directional probes	7	3	8
Radiative heat flux	5	10	11

This study provides some valuable qualitative data/insights into the effect of ventilation conditions on the fire dynamics of ISDs, particularly regarding time to flashover, conditions required for flashover and the potential fire spread to neighbouring deswellings. While the data presented in this work are not intended for direct use in quantitative risk assessments due to still need to be done repeatability work mainly to quantify the potential experimental uncertainties, they still offer a useful foundation for understanding the relative risks associated with different ventilation configurations. These conclusions can guide future studies by highlighting critical parameters that affect the fire dynamics in ISDs and their potential role in risk analysis. For instance, as shown in Table 6, the time to flashover and the HRR required for flashover could provide indications to assess the relative risk of different ISDs configurations. Cases with shorter time to flashover and lower HRR required for flashover are clearly producing higher risk, as they are more likely to contribute to rapid fire spread. In settlements with tens of thousands of dwellings, even a few seconds’ difference in time to flashover or fire spread between dwellings could significantly impact the overall rate of fire propagation across the community. This simplified presentation highlights the importance of obtaining more reliable quantitative data for parameters like time to flashover, HRR required to reach flashover and the external radiative heat fluxes to the surroundings. With such data, it would be possible to develop a more robust framework for risk-informed decision-making, ultimately enhancing the fire safety in ISs.

**Table 6:** Summary of experimental results and qualitative risk assessment for different cases

Case	Ventilation factor (m^5/2^)	${\dot{q}}_{fo}$ (MW)	Time to flashover (seconds)	Door average heat flux (kW/m^2^)	Materials in risk of ignition	Window average heat flux (kW/m^2^)	Materials in risk of ignition
BL	2.68	1.5	355	12–13	16	12–13	16
DW	3.10	2.5	350	11–12	14	10–11	9
VW	3.05	2.4	372	10–11	9		
HW	2.88	2.3	321				
BW	2.68	1.9	332			4–5	-
SRW		-	342	8–9	6		
SLW		1.7	319	7–8	4		

**Fig. 29:**
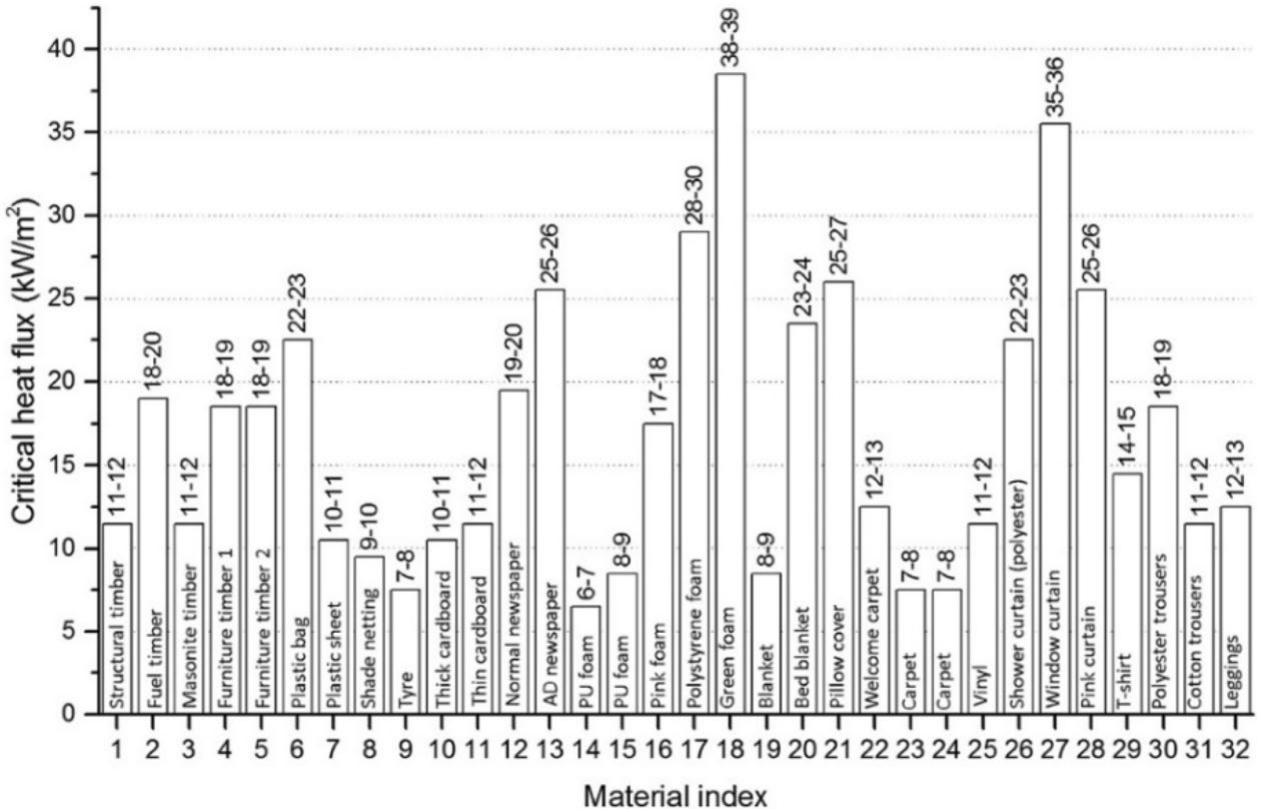
Critical heat fluxes of the 32 Martials from Wang et al. [10]

As discussed, once a structure has flashed over, the sustained flame ejection from the windows and doors could cause ignitions of nearby fuels and structures. Thus, higher heat fluxes measured implies higher risk of igniting an adjacent dwelling. By comparing the average heat fluxes at the door and window to the database of 32 materials presented by Wang et al. [10] (Fig. 29) a qualitative comparative number of materials that could be ignited can be determined and compare between ventilation cases to qualitatively understand which ventilation condition is the ‘riskiest’. From Table 6 it can be seen that the ‘riskiest’ cases in terms of fire spread post-flashover are the BL case, followed by the DW case, then the VW and HW cases. The BW case was ‘riskier’ than the side wall cases, SLW and SRW, which were found to be the ‘safest’ cases.

## Conclusions

The paper has investigated the impact of the window design and location on the fire dynamics of informal settlement dwellings by conducting six full-scale experiments in lab conditions and subsequently modelling them using the Fire Dynamics Simulator. From this paper one can conclude that:

- Placing the window on the same wall as the door leads to significantly higher measured heat fluxes along that front wall (with average of 13 kW/m^2^—2 m away from the door) compared to cases where window was located on the opposite or side walls (with averages between 7 and 11 kW/m^2^).
- Placing the window on the back wall opposite the door opening was found to produce higher heat fluxes of 11 kW/m^2^ from the door compared to 8–9 kW/m^2^ for the side wall experiments (SRW/SLW) due to the crossflow effect.
- Moving the window to a wall other than the wall with the door, slightly reduces the time to flashover, but increases the heat release rate required for flashover (by approximately 280–410 kW/m^2^, or 20–28%),
- Having two windows, one on a side wall and one on the front wall next to the door lead to one of the worst potential fire spread scenarios with average heat fluxes measured at the side wall to be around 10–11 kW/m^2^, with heat fluxes measured at the door to be around 11–12 kW/m^2^.
- Having two windows, however, did not affect the time to flashover compared to the baseline experiment but did increase the heat release rate for flashover by around 1.0 MW, or 70%.
- Doubling the size of the window (2:1 aspect ratio) and it being orientated either vertically or horizontally did not have significant effect on the heat fluxes from the window, yet slightly decreased the door’s heat fluxes due to the change in the flow dynamics through the openings, and in particular the turbulent mixing within the compartment.
- The impact on the heat release rate required for flashover was relatively unaffected by the orientation of the window openings, however the time to flashover was longer (approximately 50 s, or 13% quicker) for the tall, narrow window compared to the short, wide window.

Therefore, it has been concluded that increasing the ventilation factor by adding another window, did not reduce the heat fluxes from the front wall openings, however, it added another source of radiation to the side wall.

The work presented within this paper will be of aid engineers and urban planners in understanding and potentially mitigating urban conflagrations within these communities by providing qualitative understanding of the phenomena present within these complex structures and therefore allowing evidence-based decision making to occur.

## Supplementary Information

Below is the link to the electronic supplementary material.

Supplementary file1 (PDF 98 KB)

Supplementary file2 (PDF 549 KB)
