# Supplementary material for: Qualitative Analysis of Ventilation Position and Dimension Effects on Compartment Fire Dynamics: An Experimental and Numerical Approach
Source: Fire Technol. 2025 May 29;61(5):3615–47. doi: 10.1007/s10694-025-01747-5 (PMC12413424; doi:10.1007/s10694-025-01747-5)
Supplement: Supplementary file 1 — Supplementary file1 (PDF 98 KB) [file 10694_2025_1747_MOESM1_ESM.pdf]

AO2 CiTiceL®

with Molex connector

Performance Characteristics

**Output**	9 - 13mV in Air
**Range**	0-100% O_2
**Resolution**	0.01% O_2
**Expected Operating Life**	360000%O_2hrs at 20°C<br>286000%O_2hrs at 40°C<br>or 2 years in air at STP
**T_{90} Response Time**<br>**$T_{99.5}$ Response Time***	<5 seconds<br><40 seconds
**Signal in 100%O_2**	100±1%
**Linearity**	Linear 0-100% O_2
**Zero Offset**	<20µV
**Temperature Range**	-20°C to +50°C
**Temperature Compensation**	<2% variation from 0°C to 40°C (see graph)
**Differential Pressure Range**	0-500mbar Max
**Absolute Pressure Range**	500-2000mbar
**Relative Humidity Range**	0 to 99% non-condensing
**Long Term Output Drift**	<10% signal loss/year
**Recommended Load Resistor**	Min 10KΩ
**Warranty Period**	12 month from date of despatch

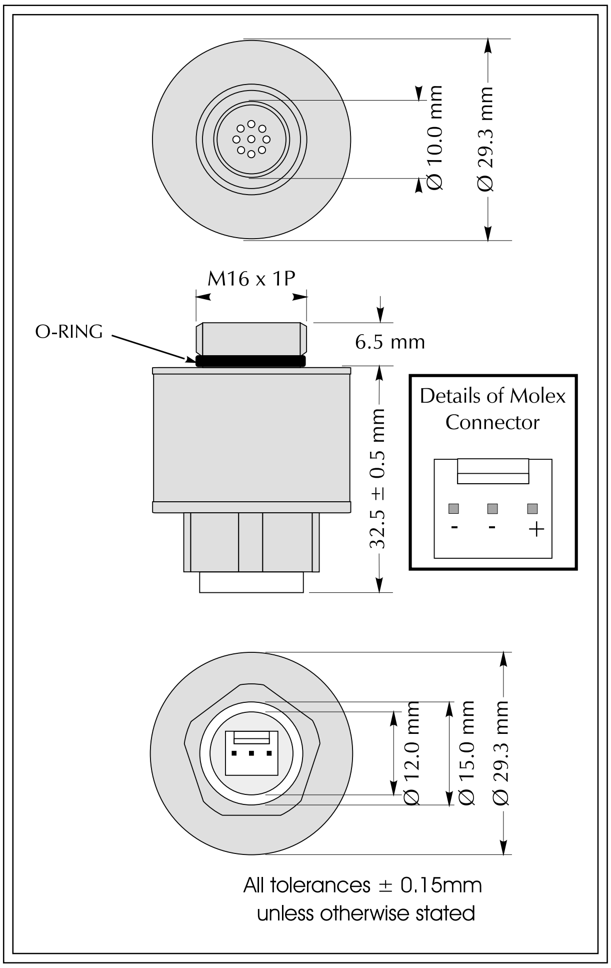

* $T_{99.5}$ response is equivalent to a change in concentration from 20.9% O_2 to 0.1% O_2

N.B. All performance data is based on conditions at 20°C, 50%RH, and 1013mBar

NOTE

Molex header used in sensor is MOLEX 22-29-2031

Suggested mating parts are:

Molex 22-01-2035: 3-way housing

Molex 08-56-0110: crimp terminals

AO2 CiTiceL to be assembled into application 'finger tight' only

City Technology Ltd, City Technology Centre, Walton Rd, Portsmouth PO6 1SZ, UK
Tel:+44 23 9232 5511, Fax:+44 23 9238 6611, sensors@citytech.co.uk, www.citytech.com

AO3 CiTiceL®

with jack socket connector

Performance Characteristics

**Output**	9 - 13mV in Air
**Range**	0-100% O_2
**Resolution**	0.01% O_2
**Expected Operating Life**	360000%O_2hrs at 20°C<br>286000%O_2hrs at 40°C<br>or 2 years in air at STP
**T_{90} Response Time**	<5 seconds
**$T_{99.5}$ Response Time***	<40 seconds
**Signal in 100%O_2**	100±1%
**Linearity**	Linear 0-100% O_2
**Zero Offset**	<20µV
**Temperature Range**	-20°C to +50°C
**Temperature Compensation**	<2% variation from 0°C to 40°C (see graph)
**Differential Pressure Range**	0-500mbar Max
**Absolute Pressure Range**	500-2000mbar
**Relative Humidity Range**	0 to 99% non-condensing
**Long Term Output Drift**	<10% signal loss/year
**Recommended Load Resistor**	Min 10KΩ
**Warranty Period**	12 month from date of despatch

* $T_{99.5}$ response is equivalent to a change in concentration from 20.9% O_2 to 0.1% O_2

N.B. All performance data is based on conditions at 20°C, 50%RH, and 1013mBar

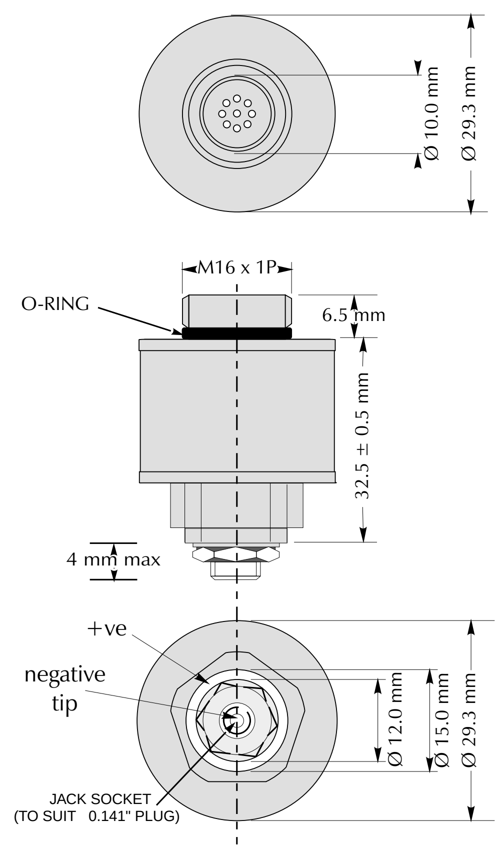

AO3 CiTiceL to be assembled into application 'finger tight' only

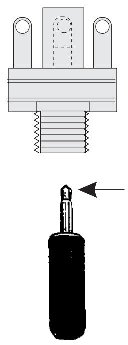

Details of Jack Socket Connection

City Technology Ltd, City Technology Centre, Walton Rd, Portsmouth PO6 1SZ, UK
Tel:+44 23 9232 5511, Fax:+44 23 9238 6611, sensors@citytech.co.uk, www.citytech.com
