# Supplementary material for: Qualitative Analysis of Ventilation Position and Dimension Effects on Compartment Fire Dynamics: An Experimental and Numerical Approach
Source: Fire Technol. 2025 May 29;61(5):3615–47. doi: 10.1007/s10694-025-01747-5 (PMC12413424; doi:10.1007/s10694-025-01747-5)
Supplement: Supplementary file 2 — Supplementary file2 (PDF 549 KB) [file 10694_2025_1747_MOESM2_ESM.pdf]

Crestline Instruments, Inc.

Model 7911

Product Manual
Automotive Infrared Gas Analyzer
(Non-dispersive Infrared Spectrometer)

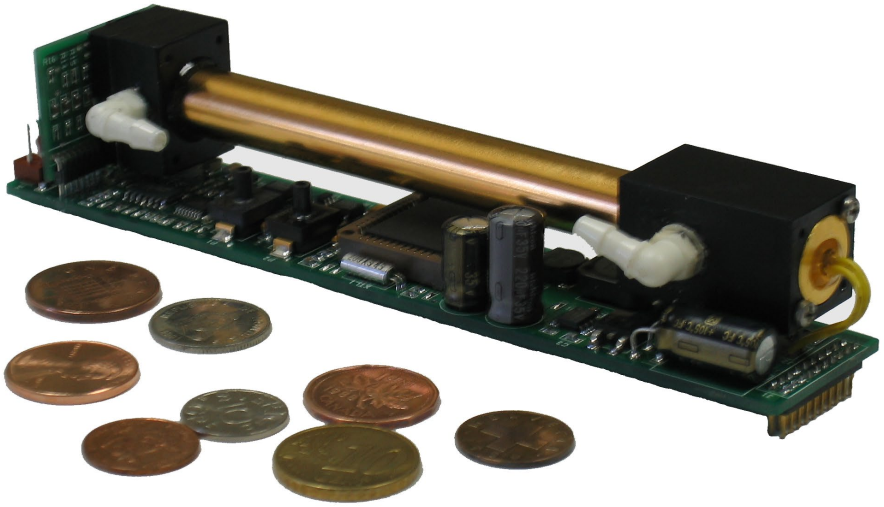

October 2010

Crestline Instruments, Inc.
2260 Crestline Road Pleasanton, CA 94566
USA

CONTENTS

I. Product Overview .... Page 1

II. 7911 Automotive Gas Analyzer Specifications .... Page 3

III. Hardware Interfaces .... Page 6

IV. Serial Host/Bench Communication Protocol .... Page 11

PRODUCT OVERVIEW

The 7911 automotive gas analyzer is offered by Crestline Instruments as a low cost OEM gas bench module or as a licensed design package that can be integrated into the product hardware architecture. With either product option, gas analyzer systems can be designed to meet the market requirements of inspection grade or repair grade equipment.

Product Features

Licensed design package:

- Ability to customize electronics to exact product requirements.
  - reduce costs
  - smaller product size
  - reduce number of cables and connectors
- Control manufacturing process of complete system.
- Provide greater service support to customers.
  - More extensive field repair capability of products
  - Reduce service inventory of product
  - Eliminate service time by gas bench vendor

Both licensed and direct purchase of 7911 OEM module provide:

- **Bench specifications for BAR-97 and OIML class 0 equipment.**
- **Three optical gas channels (HC, CO2, and CO) and analog inputs for electrochemical O2 and NO sensors.**
- **Low power consumption** (<0.75 watt)
  - Faster warm-up time
  - No fans required
    - No moving parts
    - Reduces dirt and contamination into product electronics
    - No ventilation holes required
  - No heat sinks required
  - Can use small, light weight batteries in portable products
  - Longer operating time with battery operated equipment
- **Accepts a broad voltage range of input power** (+8 to +42 VDC)

Small product volume

Compatible with portable product size and weight requirements.

Reliable Design

Low demonstrated field failures

Ancillary Product Support

Software Handlers for customer host development

Host Software

Demonstration host with following functions:

1. Numerical display of five gases, and lambda
2. Graphical display of gases
3. Data logging and disk storage capability
4. Bench zero capability
5. Bench span capability
6. EEPROM read and write capability

Sample host for user development

Calibration software

7911 AUTOMOTIVE GAS ANALYZER SPECIFICATIONS

Environmental Specifications

Temperature: Storage : -40 to +70°C ; Operating: 0 to 50°C

Humidity: Storage: 0 to 99%RH (non condensing)
Operating : 0 to 95% RH (non condensing)

Altitude: Storage: -1000 to 12000 meters; Operating: -300 to 3000 meters

Measured Gases:

**- Hydrocarbons (hexane equivalent)**	**NDIR**
**- Carbon monoxide**	**NDIR**
**- Carbon Dioxide**	**NDIR**
**- Nitric Oxide**	**Electrochemical cell**
**- Oxygen**	**Electrochemical cell**

Gas	Measuring range	Accuracy	Repeatability	Noise
**HC n-hexane**	0 to 2000ppm	±4ppm abs or ±3% rel	±3 ppm abs. or ±2 % rel	2ppm abs or 0.8% rel
**HC Propane**	0 - 4000 ppm	±8ppm abs or 3% rel	±6 ppm abs or 2% rel	4ppm abs or 0.8% rel
	4001 - 10000ppm	±5% rel	±3 % rel	
	10001 - 30000ppm	±10% rel	±5% rel	
**CO**	0.00% to 10.0%	±0.02%abs or ± 3% rel	±0.02 abs or ±2% rel	0.01% abs or 0.8% rel
	10.01% to 15.0%	±5% rel	±3% rel	0.8% rel
**CO_2**	0.00% to 16%	±0.3% abs or ±3% rel	±0.1% abs or ±2% rel	0.1% abs or 0.8% rel
	16.01% to20.00%	±5% rel	±3% rel	or 2% rel
**NO**	0 to 5000ppm	±5ppm abs or 1% rel	±5 ppm abs or 1% rel	5ppm abs or 1% rel
**0_2**	0.00 to 25.00%	±0.02%abs or 1% rel	±0.02% abs or 1%rel	0.02% abs or 1%rel

Response time:

**T_{90}**	Less than 3.5 seconds for HC, C0 and CO_2
**T_{95}**	Less than 4.5 seconds for HC C0 and $C0_2$
**T_{10}**	Less than 3.7 seconds for HC, C0 and $C0_2$
**T_5**	Less than 4.7 seconds for HC, C0 and $C0_2$

Flow rate: minimum = 0.5 liters/minute maximum = 1.5 liters/minute

Cross Gas Interference:

Test gases	Maximum cross gas interference fromany test gas
16% CO_2 in nitrogen	
1600 ppm HC in nitrogen	
10% CO in nitrogen	±4 ppm HC, n-hexane equivalent
3000ppm NO in nitrogen	±0.02% CO
75 ppm H_2S in nitrogen	±0.2 % CO_2
75 ppm SO_2 in nitrogen	±20 ppm NO
18% CO_2 and 9% CO in nitrogen	
Water saturated air at 50 °C	

Warm-up time: Full accuracy in two minutes

Electrical Specifications:

Power consumption: <0.7 watt average power
<1.5 watt peak power

Input voltage range: 8- 42VDC

Input regulation: Unregulated DC between 8 and 42 VDC

Mechanical Specifications:

Dimensions: 146 mm L x 30mm W x 33 mm H
Weight: 75 grams

User Interface

Host Communication Interface:

Interface type: RS232, asynchronous
Baud rate: 9600bps
Format: 1 start bit, 8 data bits, no parity bit, 1 stop bit
Signals: Transmit data, receive data, signal ground

Auxiliary I/O:

Input signals: Tachometer input, 0-5 V pulse. Times between leading edges. Three analog signals. 0- 5.000 VDC. 8-bit ADC resolution.

Outputs: 5.4 VDC regulated, up to 2 Watts. Flow occlusion Signal**.**

Eight user defined CMOS outputs and digital ground. All outputs are terminated with a 10K ohm pull down resistor.

Miscellaneous

The 7911 gas bench meets or exceeds all measurement requirements to comply with BAR-97 and OIML class 0 inspection equipment.

Note 1: In all gas accuracy, repeatability and noise specifications the greater of the absolute or the relative value is the applicable tolerance

***Note 2*:** For NO and O_2 specifications the gas accuracy, repeatability and noise specifications are the contribution of the model 7911 signal processing circuits. The customer has the responsibility to provide NO and O_2 sensors with the desired performance characteristics.

HARDWARE INTERFACES

Mechanical Data

Physical Characteristics

Weight	75 g
Length	146mm
Width	30mm
Height	33mm
Recommended Storage Temperature Range	-40 to 70ºC

Outline Dimensions

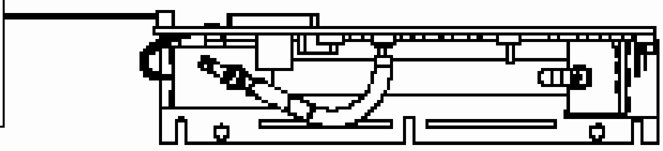

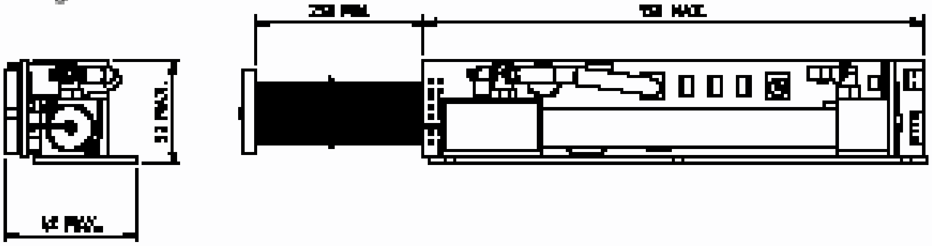

Electrical Connectors

Connector J1 **Main Connector**

The main connector is marked J1 and is a 2 x 10 header with 2 mm spaced centers. This connector is used to deliver power to the bench, establish serial communications,

Signal	Pin	Pin	Signal
VPWR	19	20	VPWR
TACH			TXD#
USER7			RXD#
USER3			Vcc
USER6			USER5
USER4			USER2
USER1			USER0
EXTA3			EXTINT
EXTA2			EXTA1
GND	1	2	GND

**Pin 1, 2**	**GND**	Ground. Good grounding practices are important for proper operation of the 7911 bench.

Analog Input Signals:
Three analog channels are available for user system signals. These channels are intended for use with analog signals having T_{10} to T_{90} response time requirements of greater than 1 second. Typical uses for these analog channels are for oil temperature, tilt sensors, and filter liquid level sensing.

**Pin 3**	**EXTA2**	User analog signal, 0 to 5 volts
**Pin 4**	**EXTA1**	User analog signal, 0 to 5 volts.
**Pin 5**	**EXTA3**	User analog signal, 0 to 5 volts.
**Pin 6**	**EXTINT**	Gas flow occlusion. The vacuum transducer included on the 7911 bench is typically located at the input of the pneumatic pump in order to detect a increased vacuum when the pneumatic lines are blocked. The vacuum signal is measured at 2 msec. intervals and compared with the threshold value set by the user. If the vacuum signal exceeds the threshold value, EXTINT goes high. When the vacuum signal decreases below the designated threshold, EXTINT is set low. The threshold is set using the ***SET THRESHOLD*** command. Details on setting the vacuum threshold are given in the Host Command Protocol section.

Pins 7, 8, 9, 10, 11, 12, 13, and 15 are user control signals. They can be set high or low using the ***CONTROL*** command described in the Host Command Protocol. An additional feature of these pins is that they can be set to any desired state when the vacuum transducer signal exceeds the threshold level. The state of each of the eight control signals is determined by the user control mask that is defined using the ***SET USER MASK*** command. This command is described in the Host Command Protocol.

**Pin 14**	**Vcc**	The input power (8 to 42VDC) is converted to +5.4V using a buck switching regulator. Two watts of power at +5.4 volts is available for ancillary system use.

**Pin 17**	**TACH**	TACH is an input signal (0 to 5V) that measures the time interval between the leading edges of two input pulses. The time interval can range from 3msec. to 300 msec. The time interval is reported in increments of 0.5 microseconds. A tachometer pulse stream of 100 Hz (6000 rpm) would therefore have a reported value from the data stream of 20000.

Data Communication (Pins 16 and 18):

The 7911 gas bench receives commands from a host computer using an RS-232 serial interface. Communication is performed using the following hardware protocol:

Baud rare:	9600
Format:	1 start bit, 8 data bits, no parity bit, 1 stop bit
Signals:	Transmit data, receive data, signal ground

Host commands are available to request data, control I/O signals, and calibrate the bench. Details of the host communication software protocol are provided in section IV.

**Pin 16**	**RXD#**	Pin 16 is the RS-232 serial input line
**Pin 18**	**TXD#**	TXD is the RS-232 output line

Input Power (Pins 19 and 20):

**Pins 19, 20**	**VPWR**	System input power (8 to 42 VDC). The 7911 gas bench contains an efficient switching buck regulator to accept unregulated DC power ranging from 8 volts to 42 volts.

WARNING -- Reverse polarity protection is not provided and must be supplied by the user if reverse polarity conditions are possible. Damage to the bench will occur with reverse polarity power.

Connector J3, Oxygen sensor connector.

1 **Ground**

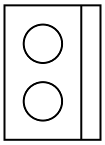

2 **0 to 12mV signal**

The oxygen sensor is provided to enable the measurement of oxygen with volumetric concentrations ranging from 0 to 25%. Oxygen in automotive exhaust gas is typically measured using an electrochemical cell that generates <12mV in the presence of 20.9% O2. This channel is ESD protected with zener diodes. A pull down resistor is also included to insure a zero signal when the oxygen sensor is disconnected.

The oxygen input signal is amplified by the bench electronics before connecting to a 12 bit analog to digital converter. An RC filter is also included to reduce noise that has a T_{10} to T_{90} response time of 2 seconds. The bench uses low offset and low drift amplifiers to condition the signal for the ADC. However, due to the high gain of this circuit, some residual zero offset may be observed. Using the ***O2 ZERO*** command, this offset is eliminated by storing its value in EEPROM and making a software correction. The ***O2 ZERO*** command is described in the software protocol section.

Over time, the output voltage from an electrochemical oxygen sensors will decrease with a given concentration of O2. During ***BENCH ZERO*** operations, the electrochemical oxygen cell is calibrated with room air that is assumed to contain 20.8% oxygen. The 7911 bench sets a warning bit if the input signal is less than 7mV during a zero operation. In addition, if the signal is less than 5mV during the BENCH ZERO operation, an error bit is set by the system software. The 5mV error bit default can be changed using the **O2 SENSOR FAILURE LIMIT** command. This command is described in the software protocol section. Details of oxygen error conditions are given in the software protocol section.

NOTE: The BENCH ZERO command checks for a valid oxygen signal before executing the zero operation. The zero operation will be ignored if the oxygen signal is not within the proper system limits (7mV (default) to 12mV with 20.9% oxygen).

Connector J4, NO Electrochemical Sensor

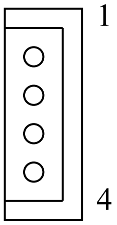

Ground
+5V
-5V
NO input signal (0 to 5 volts).

Connector J4 is used to measure the concentration of NO from an external sensor that produces a signal (0 to 5V) that is linearly proportional to NO concentration. This channel has no signal conditioning prior to input to a 10 bit analog to digital converter. The maximum NO gas concentration should therefore produce a signal slightly less than 5V to obtain the maximum resolution from the ADC. Calibration of this signal is performed using the ***BENCH SPAN*** command. Any zero offset is eliminated with

software corrections in conjunction with the ***BENCH ZERO*** command. The signal is reported to the host computer using the ***COMPENSATED DATA*** command. These host/bench commands are described in the host software protocol section.

If nitric oxide measurements are not required in the user's product, the NO channel can be used for any other system signal with an input range of 0 to +5V.

+5V, -5V, are provided for NO sensor subsystem electronics. The +5V and –5V power rails can be used for any system electronic circuits **provided that no more than 25mA are used from each of these power rails**.

Pneumatic Connections:

The 7911 gas bench is designed to operate to specifications with a volumetric flow rate of 0.5 to 1.5 liters per minute. Reduced flow rates can increase the response time of gas measurements. Inlet and outlet gas connections are made with hose-barb fittings. To reduce the adsorption of hydrocarbons in the sample train, it is recommended that tubing, connections, filters and pump internal components be constructed of low hydrocarbon affinity materials such as PTFE, viton or polyurethane.

The vacuum transducer has a 2.8 mm diameter port fitting. To avoid oscillating signals due to pump compression cycles, it is recommended that a small orifice be placed between the pneumatics line being monitored and the transducer. The smaller the orifice diameter, the quieter the signal. Larger diameter orifices will give faster response to vacuum transients. The volume between the orifice and the vacuum transducer will also influence the pulse dampening and response time.

SERIAL HOST/BENCH COMMUNICATION PROTOCOL

The 7911 gas bench operates in conjunction with the gas analyzer system computer by means of an RS-232 serial communications link. For reliable operation, commands, data, and status information must be properly formatted. This section describes the details of the communication protocol that enable the user to send commands to the bench, receive data from the bench, and check the operating status of the 7911 unit.

Each command string from the host, and each response from the gas monitor must start with the STX character (ASCII 02), and terminate with a checksum (See checksum data section). In addition, all responses from the bench will include status data (see Status data section) immediately preceding the checksum. The serial interface will remain inactive until a start of transmission character (ASCII $02) is received. The STX character should be followed by a command character and any data needed by the specified command. Finally, a checksum is required to complete the command sequence.

Once a command sequence has been sent from the host, no other commands should be sent until a response is received from the analyzer. If no response is received from the analyzer before the time-out specification for a command, one can assume that no response will be given. After the time-out period, further commands can be issued from the host system.

If the command sequence was improperly formatted, or a transmission error occurred, the error response sequence will be returned by the bench. This consists of the NAK identifier ($15), the bench status and checksum.

The communication protocol is designed to accept ASCII characters, 8 bit binary, 16 bit binary, 24 bit binary data. All ASCII characters are transmitted as 7-bit ASCII characters. If the eighth bit is set, the data is assumed to be a binary format as described in the following table.

Data Formats for 7911 Communication Protocol

Type of Data	High order nibble	Comments
7 bit ASCII character	$0 to $7	All printable characters ($20 to $1F)
8 bit binary data	$8	Sent as two bytes.<br>e.g. $2A sent as:<br>$82 $8A
16 bit binary data	$9	Sent as four bytes.<br>e.g. $BD2A sent as<br>$9B $9D $92 $9A
24 bit binary data	$A	Sent as six bytes.<br>e.g. $4CBD2A sent as<br>$A4 $AC $AB $AD $A2 $AA
Status data	$C<br>$B	ST1 – Most significant 4 bits<br>ST2 – Least significant 4 bits<br>e.g. Status $C2 sent as $CC $B2
Checksum data	$E<br>$D	CS1 – Most significant 4 bits<br>CS2 – Least significant 4 bits<br>e.g. Checksum $8A sent as $E8 $DA

Commands sent from the host system to the gas analyzer have different string lengths depending on the amount of information required. The command list is given below. Details for each command are given in the following section.

Command Summary

Command Name	Hex Cha r	Description or Comments
RESET	$30	Restart analyzer software
COMPENSATED DATA	$31	Report compensated gas data & tach.
ENVIRONMENTAL DATA	$32	Report press, flow, temp, AD-1, AD-2
RAW DATA #1	$33	Report raw uncompensated data
RAW DATA #2	$34	Report raw data continued.
ZERO BENCH	$35	Zero the analyzer channels
BENCH SPAN	$36	Span the designated data channels.
PEF CALULATION	$37	Report PEF at a given propane conc.
SPAN RESET	$38	Reset designated channels to factory
READ EEPROM BYTE	$39	Read designated EEPROM location
READ EEPROM BLOCK	$3A	Read a block of EEPROM data
WRITE EEPROM BYTE	$3B	Write data to designated EEPROM loc.
WRITE EEPROM BLOCK	$3C	Write a block of EEPROM data
READ BENCH DATA	$3D	Read firmware version; SN; and PEF
READ SERVICE STATUS BITS	$3E	Read service EEPROM failure codes
VACUUM TRANSDUCER THRESHOLD	$3F	Set the flow interrupt threshold level
ZERO FLOW CHANNEL	$40	Zero the flow channel zero offset (pump off)
CONTROL SIGNALS	$41	Set user control signals
ZERO O2	$42	Zero O2 channel
CLEAR O2 ERROR BIT	$43	Clear the O2 error bit in EEPROM
CLEAR NO ERROR BIT	$44	Clear the NO error bit in EEPROM
SET USER I/O MASK	$45	Set I/O mask for flow fault
READ USER I/O STATES	$46	Read state of User I/O lines.
ZERO REQUEST INTERVAL	$47	Set bench zero request interval after warm-up
DEVICE ID	$48	Report analyzer type to system.
DOWNLOAD SOFTWARE	$49	Sends new embedded code to analyzer
READ BENCH OPERATING STATUS	$4A	Read operating condition status bits
EXTENDED COMPENSATED DATA	$4B	Report 5 gas data, pressure, temp, tach, status
O2 SENSOR FAILURE LIMITS	$4C	Set failure limits for O2 input signal.
METHANE/PROPANE MODE CONTROL	$4D	Sets HC display to methane or propane
LAMP ON/OFF	$4E	Sets bench in standby mode with lamp off

Command Details

$30 RESET

The reset command enables the user to restart the 7911 bench without turning off the input power. This command is useful for clearing status conditions which might arise from abnormal conditions such as temperatures exceeding product specifications.

**Command**	**$02**<br>**$30**<br>**$E3 $D0**	**ASCII**<br>**ASCII**<br>**Checksum**	**STX character**<br>**Command character**<br>**Checksum**
**Response**	**$02**<br>**$30**<br>**ST1 ST2**<br>**CS1 CS2**	**ASCII**<br>**ASCII**<br>**Status**<br>**Checksum**	**STX character**<br>**Command character**<br>**Status information**<br>**Checksum**
**Timeout interval**	**2 seconds**		

$31 COMPENSATED DATA

The compensated data command enables the user to receive concentrations for Hex, Pro, CO, CO2, O2, NOx and tachometer readings. Data are represented by the following data types and converted to hexadecimal formats for transmission according to the protocol rules.

**Command**	**$02**<br>**$31**<br>**$E3 $D1**	**ASCII**<br>**ASCII**<br>**Checksum**	**STX character**<br>**Command character**<br>**Checksum**
**Response**	**$02**<br>**$31**<br>**hex1 hex2 hex3 hex4**<br>**pro1 pro2 pro3 pro4**<br>**CO21 CO22 CO23 CO24**<br>**CO1 CO2 CO3 CO4**<br>**O21 O22 O23 O24**<br>**NOx1 NOx2 NOx3 NOx4**<br>**Tach6 Tach5 Tach4 Tach3**<br>**Tach2 Tach1**<br>**ST2 ST1**<br>**CS1 CS2**	**ASCII**<br>**ASCII**<br>**16bit-signed**<br>**16bit-signed**<br>**16bit-signed**<br>**16bit-signed**<br>**16bit-signed**<br>**16bit-signed**<br>**24bit-unsigned**<br><br>**Status**<br>**Checksum**	**STX character**<br>**Command character**<br>**Ppm hexane**<br>**Ppm propane**<br>**Vol % CO2 x 100**<br>**Vol % CO x 1000**<br>**Vol % O2 x 100**<br>**Ppm Nitric oxide**<br>**Tachometer time**<br>**interval sec x 2,000,000**
**Timeout interval**	**2 seconds**		

$32 ENVIRONMENTAL DATA

The environmental data command is intended to query data that changes slowly and data that is not often required by the host. The parameters transmitted are sample cell pressure, gas sample flow rate, sample cell temperature, external A/D-1, external A/D-2, and external A/D-3. Each channel is represented by the following data types and are transmitted according to the protocol rules.

**Command**	**$02**<br>**$32**<br>**$E3 $D2**	**ASCII**<br>**ASCII**<br>**Checksum**	**STX character**<br>**Command character**<br>**Checksum**
**Response**	**$02**<br>**$32**<br>**P1 P2 P3 P4**<br>**T1 T2 T3 T4**<br>**F1 F2**<br>**X11 X12**<br>**X21 X22**<br>**X31 X32**<br>**X41 X42**<br>**ST1 ST2**<br>**CS1 CS2**	**ASCII**<br>**ASCII**<br>**16-bit unsigned**<br>**16-bit unsigned**<br>**8-bit unsigned**<br>**8-bit unsigned**<br>**8-bit unsigned**<br>**8-bit unsigned**<br>**8-bit unsigned**<br>**Status**<br>**Checksum**	**STX character**<br>**Command character**<br>**Pressure (kPa x 100)**<br>**Temperature (Kelvin x 10)**<br>**Flow kPa gauge**<br>**Ext. analog 1 (volts)**<br>**Ext. analog 2 (volts)**<br>**Ext. analog 3 (volts)**<br>**Reserved (ground)**<br>**Status information**<br>**Checksum**
**Timeout interval**	**2 seconds**		

$33 RAW DATA-1

The raw data command is intended to provide the primitive signal levels used to calculate concentrations. This command is primarily used for diagnostics.

**RAW DATA-1 Command**	**$02**<br>**$33**<br>**$E3 $D3**	**ASCII**<br>**ASCII**<br>**Checksum**	**STX character**<br>**Command character**<br>**Checksum**
**RAW DATA-1 response**	**$02**<br>**$33**<br>**C01 C02 C03 C04**<br>**C11 C12 C13 C14**<br>**C21 C22 C23 C24**<br>**C31 C32 C33 C34**<br>**C41 C42 C43 C44**<br>**C51 C52 C53 C54**<br>**C61 C62 C63 C64**<br>**C71 C72 C73 C74**<br>**tach1 tach2 tach3**<br>**tach4 tach5 tach6**<br>**ST1 ST2**<br>**CS1 CS2**	**ASCII**<br>**ASCII**<br>**16-bit unsigned**<br>**16-bit unsigned**<br>**16-bit unsigned**<br>**16-bit unsigned**<br>**16-bit unsigned**<br>**16-bit unsigned**<br>**16-bit unsigned**<br>**16-bit unsigned**<br>**24-bit unsigned**<br><br>**Status**<br>**Checksum**	**STX character**<br>**Command character**<br>**HC signal (counts)**<br>**CO2 signal (counts)**<br>**CO signal (counts)**<br>**Reference signal (counts)**<br>**Temperature (counts)**<br>**O2 (counts)**<br>**NOx (counts)**<br>**Pressure (counts)**<br>**Tachometer time interval**<br>**.5 µsec/count**<br>**Status information**<br>**Checksum**
**Timeout**	**2 seconds**		

$34 RAW DATA-2

The raw data-2 command is intended to provide the primitive signal levels used by the analyzer to calculate flow, ambient temperature, external A/D-1, external A/D-2, and +5 volt power. This command is used primarily for diagnostics.

**Command**	**$02**<br>**$34**<br>**$E3 $D4**	**ASCII**<br>**ASCII**<br>**Checksum**	**STX character**<br>**Command character**<br>**Checksum**
**Response**	**$02**<br>**$34**<br>**MX01 MX02**<br>**MX11 MX12**<br>**MX21 MX22**<br>**MX31 MX32**<br>**MX41 MX42**<br>**MX51 MX52**<br>**MX61 MX62**<br>**MX71 MX72**<br>**ST1 ST2**<br>**CS1 CS2**	**ASCII**<br>**ASCII**<br>**8-bit unsigned**<br>**8-bit unsigned**<br>**8-bit unsigned**<br>**8-bit unsigned**<br>**8-bit unsigned**<br>**8-bit unsigned**<br>**8-bit unsigned**<br>**8-bit unsigned**<br>**Status**<br>**Checksum**	**STX character**<br>**Command character**<br>**Reserved (ground)**<br>**5 * C^o**<br>**Ext. analog 2 (counts)**<br>**Flow (counts)**<br>**+5V (counts)**<br>**Ext. analog 3 (counts)**<br>**Ext. analog 1 (counts)**<br>**Reserved (ground)**<br>**Status information**<br>**Checksum**
**Timeout**	**2 seconds**		

$35 ZERO

The zero command enables the user to adjust calibration factors so that the analyzer reports zero concentration for HC, CO, CO2 and NOx. The zero command assumes that room air is used during this calibration process. The Oxygen concentration is spanned during this command and is calibrated to 20.9% oxygen by volume. If the oxygen signal is not greater than 3mV (or the value set using the ***O2 SENSOR FAILURE LIMITS*** command) during the zero command, the zero will be aborted and the zero request bit in the STATUS byte will remain set.

**Command**	**$02**<br>**$35**<br>**$E3 $D5**	**ASCII**<br>**ASCII**<br>**Checksum**	**STX character**<br>**Command character**<br>**Checksum**
**Response**	**$02**<br>**$35**<br>**ST1 ST2**<br>**CS1 CS2**	**ASCII**<br>**ASCII**<br>**Status**<br>**Checksum**	**STX character**<br>**Command character**<br>**Status information**<br>**Checksum**
**Timeout**	**15 seconds**		

$36 SPAN SPECIFIED CHANNELS

The span command enables the user to calibrate each gas channel. Gas calibration is achieved by designating the gas channel desired, and the concentration (tag value) of the standard gas sample used.

**Command**	**$02**<br>**$36**<br>**CH1 CH2**<br>**TV1A TV2A TV3A TV4A**<br>**TV1B TV2B TV3B TV4B**<br>**TV1C TV2C TV3C TV4C**<br>**TV1D TV2D TV3D TV4D**<br>**TV1E TV2E TV3E TV4E**<br>**CS1 CS2**	**ASCII**<br>**ASCII**<br>**8 bit**<br>**16 bit**<br>**16 bit**<br>**16 bit**<br>**16 bit**<br>**16 bit**<br>**checksum**	**STX character**<br>**Command character**<br>**Gas channels**<br>**ppm hexane**<br>**ppm propane**<br>**Percent CO2 x 100**<br>**Percent CO x 1000**<br>**ppm NO**<br>**Checksum**
**Response**	**$02**<br>**$36**<br>**ST1 ST2**<br>**CS1 CS2**	**ASCII**<br>**ASCII**<br>**Status**<br>**Checksum**	**STX character**<br>**Command character**<br>**Status information**<br>**Checksum**
**Timeout**	**20 seconds**		

$37 CALCULATE PEF VALUE

The ***Calculate PEF*** command allows the user to obtain a ***propane equivalency factor*** for any concentration of propane used as a calibration standard. The propane equivalency factor is a multiplier to convert reported propane concentrations by the analyzer to hexane concentrations. This command is required since the response ratio between propane and hexane changes with the concentration of propane and different propane concentrations are used as calibration standards.

Note: The PEF command is required only once if the same propane concentration is always used for calibration. The PEF value can be read from EEPROM using the *BENCH DATA* command.

PEF COMMAND

**Command**	**$02**<br>**$37**<br>**TV1 TV2 TV3 TV4**<br>**$CS1 $CS2**	**ASCII**<br>**ASCII**<br>**Checksum**	**STX character**<br>**Command character**<br>**Checksum**
**Response**	**$02**<br>**$37**<br>**PEF1 PEF2 PEF3 PEF4**<br>**ST1 ST2**<br>**CS1 CS2**	**ASCII**<br>**ASCII**<br>**16-bit unsigned**<br>**Status**<br>**Checksum**	**STX character**<br>**Command character**<br>**PEF value x 1000**<br>**Status information**<br>**Checksum**
**Timeout**	**2 seconds**		

$38 SPAN RESET DESIGNATED CHANNELS

The span reset command is provided to reset the customer span factors to the factory default values. This command clears span error bits in EEPROM and sets the designated gas channel span factor to 1.00.

**Command**	**$02**<br>**$38**<br>**CH1 CH2**<br>**CS1 CS2**	**ASCII**<br>**ASCII**<br>**8-bit**<br>**Checksum**	**STX character**<br>**Command character**<br>**Gas channel(s)**<br>**Checksum**
**Response**	**$02**<br>**$38**<br>**ST1 ST2**<br>**CS1 CS2**	**ASCII**<br>**ASCII**<br>**Status**<br>**Checksum**	**STX character**<br>**Command character**<br>**Status information**<br>**Checksum**
**Timeout**	**2 seconds**		

**CH bit 0**	**Hexane span reset**
**CH bit 1**	**Propane span reset**
**CH bit 2**	**CO2 span reset**
**CH bit 3**	**CO span reset**
**CH bit 4**	**Nitric oxide span reset**
**CH bits 5-7**	**Reserved**

$39 READ EEPROM BYTE

The Read EEPROM command is intended to provide the user with the ability to read all values of EEPROM. This command is useful to inspect bench calibration values, serial numbers, and user information.

**Command**	**$02**<br>**$39**<br>**Loc1 Loc2 Loc3 Loc4**<br>**CS1 CS2**	**ASCII**<br>**ASCII**<br>**16 bit**<br>**Checksum**	**STX character**<br>**Command character**<br>**EEPROM address**<br>**Checksum**
**Response**	**$02**<br>**$39**<br>**VH VL**<br>**ST1 ST2**<br>**CS1 CS2**	**ASCII**<br>**ASCII**<br>**8 bit**<br>**Status**<br>**Checksum**	**STX character**<br>**Command character**<br>**Value**<br>**Status information**<br>**Checksum**
**Timeout**	**2 seconds**		

$3A READ EEPROM BLOCK

The Read EEPROM block command enables the user to read large segments of EEPROM data in a single block transfer. The maximum block count is 256 bytes.

**Command**	**$02**<br>**$3A**<br>**Loc1 Loc2 Loc3 Loc4**<br>**Count1 Count2**<br>**CS1 CS2**	**ASCII**<br>**ASCII**<br>**16 bit**<br>**8 bit**<br>**Checksum**	**STX character**<br>**Command character**<br>**Start location**<br>**Number of bytes**<br>**Checksum**
**Response**	**$02**<br>**$3A**<br>**V1H V1L**<br>**V2H V2L**<br>**…….**<br>**VnH VnL**<br>**ST1 ST2**<br>**CS1 CS2**	**ASCII**<br>**ASCII**<br>**8 bit**<br>**8 bit**<br><br>**8 bit**<br>**Status**<br>**Checksum**	**STX character**<br>**Command character**<br>**Value 1**<br>**Value 2**<br><br>**Value n**<br>**Status information**<br>**Checksum**
**Timeout interval**	**4 seconds**		

The following conditions will cause a NAK response from the READ EEPROM BLOCK command:

1. The base address is greater than 255.
2. The base address + block count is greater than 255. Status bit 5 will be set.

$3B WRITE EEPROM BYTE

The Write EEPROM command is intended to provide the user with the ability to save data in the analyzer EEPROM. This command is useful to save any type of information important to the user during operation such as calibration values for external analog input channels.

**Command**	**$02**<br>**$3B**<br>**Loc1 Loc2 Loc3 Loc4**<br>**VH VL**<br>**CS1 CS2**	**ASCII**<br>**ASCII**<br>**16 bit**<br>**8 bit**<br>**Checksum**	**STX character**<br>**Command character**<br>**EEPROM address**<br>**Value**<br>**Checksum**
**Response**	**$02**<br>**$3B**<br>**Loc1 Loc2 Loc3 Loc4**<br>**V1H V1L**<br>**ST1 ST2**<br>**CS1 CS2**	**ASCII**<br>**ASCII**<br>**16 bit**<br>**8 bit**<br>**Status**<br>**Checksum**	**STX character**<br>**Command character**<br>**Location**<br>**Value**<br>**Status information**<br>**Checksum**
**Timeout interval**	**2 seconds**		

$3C WRITE EEPROM BLOCK

The Write EEPROM block command enables the user to store multiple bytes of EEPROM data in a single block transfer. If while writing to the location the value is not stored correctly, the EEPROM write failure bit (bit3) in the extended EEPROM failure codes (byte0) is set. The value returned is then the incorrect value stored and the location is the address where the error occurred. A maximum of 16 EEPROM locations can be written by one execution of the WRITE EEPROM BLOCK command. (The command will be ignored if more than 40 bytes are sent to the gas bench).

**Command**	**$02<br>$3C<br>Loc1 Loc2 Loc3 Loc4<br>V0H V0L<br>V1H V1L<br>……..<br>VnH VnL<br>CS1 CS2**	**ASCII<br>ASCII<br>16 bit<br>8 bit<br>8 bit<br><br>8 bit<br>Checksum**	**STX character<br>Command character<br>Location<br>Value 0<br>Value 1<br><br>Value n<br>Checksum**
**Response (successful)**	**$02<br>$3C<br>ST1 ST2<br>CS1 CS2**	**ASCII<br>ASCII<br>Status<br>Checksum**	**STX character<br>Command character<br>Status information<br>Checksum**
**Response (unsuccessful)**	**$02<br>$3C<br>Loc1 Loc2 Loc3 Loc4<br>VH VL<br>ST1 ST2<br>CS1 CS2**	**ASCII<br>ASCII<br>16 bit<br>8 bit<br>Status<br>Checksum**	**STX character<br>Command character<br>Location<br>Value<br>Status information<br>Checksum**
**Timeout**	**4 seconds**		

EEPROM Notes

The 7911 bench has 256 bytes of EEPROM memory that are used for storing data. The first 73 locations (0-72) of the EEPROM are available to the user for data storage. Locations 73 to 255 are used by the 7911 operating system and are protected from use by the host system.

$3D BENCH DATA

The Bench Data command is designed to provide information to the user that is not related to gas measurements. Parameters reported in the bench data command are:

Software revision.
Serial Number.
Date of manufacture.
Vacuum threshold.
Extended error status (service status bits).
Propane equivalence factor (PEF).
The propane concentration from which the PEF value is calculated.

**Command**	**$02**<br>**$3D**<br>**$E3 $DD**	**ASCII**<br>**ASCII**<br>**Checksum**	**STX character**<br>**Command character**<br>**Checksum**
**Response (successful)**	**$02**<br>**$3D**<br>**REVA REVB**<br>**SN1 SN2 SN3 SN4**<br>**SN5 SN6 SN7**<br>**MD1 MD2 MD3 MD4**<br>**MD5 MD6 MD7 MD8**<br>**MD9 MD10 MD11 MD12**<br>**TH1 TH2**<br>**V0H V0L**<br>**V1H V1L**<br>**V2H V2L**<br>**V3H V3L**<br>**PEF PEF PEF PEF**<br>**PTAG PTAG PTAG PTAG**<br>**ST1 ST2**<br>**CS1 CS2**	**ASCII**<br>**ASCII**<br>**ASCII**<br>**ASCII**<br>**ASCII**<br>**ASCII**<br>**ASCII**<br>**ASCII**<br>**8 bit**<br>**8 bit**<br>**8 bit**<br>**8 bit**<br>**8 bit**<br>**16 bit**<br>**16 bit**<br>**status**<br>**checksum**	**STX character**<br>**Command character**<br>**Software revision**<br>**Bench serial number**<br><br>**Date of manufacture**<br><br><br>**Vacuum threshold**<br>**Service error byte 0**<br>**Service error byte 1**<br>**Service error byte 2**<br>**Service error byte 3**<br>**PEF x 1000**<br>**Propane conc. (ppm)**
**Timeout**	**2 seconds**		

The serial number can be sent as 7 or 8 digits depending on the value of bit 0 of byte F4 in EEPROM. If true, an eight byte serial number is reported. If false, a seven byte serial number is reported. This feature is available to provide compatibility for legacy software with the IRidium 100 bench manufactured by City Technology Ltd. The default is bit 0 = 0. Crestline will configure the software for 8 byte serial numbers upon request.

$3E READ SERVICE EEPROM FAILURE CODES

The 7911 bench constantly checks for error conditions during measurement operations. If errors occur that compromise the integrity of the data, error bits will be set. Hardware errors, and span errors are recorded in 4 bytes of EEPROM so that diagnostics can be performed on the analyzer during service operations. The Read Extended EEPROM error codes command allows the service technician to conveniently access the hardware error bits which are stored in EEPROM**. Errors are latched in the extended error bytes. This data is intended for service diagnostics.** For run time errors and status conditions, see command $4A.

**Command**	**$02**<br>**$3E**<br>**$E3 $DE**	**ASCII**<br>**ASCII**<br>**Checksum**	**STX character**<br>**Command character**<br>**Checksum**
**Response**	**$02**<br>**$3E**<br>**V0H V0L**<br>**V1H V1L**<br>**V2H V2L**<br>**V3H V3L**<br>**ST1 ST2**<br>**CS1 CS2**	**ASCII**<br>**ASCII**<br>**8 bit**<br>**8 bit**<br>**8 bit**<br>**8 bit**<br>**Status**<br>**Checksum**	**STX character**<br>**Command character**<br>**Error byte 0**<br>**Error byte 1**<br>**Error byte 2**<br>**Error byte 3**<br>**Status information**<br>**Checksum**
**Timeout interval**	**2 seconds**		

Error Codes

Byte	Bit	Meaning
0	0	0 = PASS 1 = FAIL RAM read/write test.
	1	0 = PASS 1 = FAIL ROM checksum test
	2	Watchdog timeout
	3	EEPROM write failure.
	4	Reference channel light level below operation specifications.
	5	Low HC light level during ZERO command.
	6	Low CO2 light level during ZERO command.
	7	Low CO light level during ZERO command.
1	0	Reference channel ADC out of range.
	1	O2 channel ADC out of range.
	2	HC channel ADC out of range.
	3	NO channel ADC out of range.
	4	CO2 channel ADC out of range.
	5	Sample cell temperature ADC out of range.
	6	CO channel ADC out of range.
	7	Pressure channel ADC out of range.
2	0	Ground fault.
	1	Ambient temperature out of range.
	2	EXTA2 channel error.
	3	Flow channel error.
	4	Power supply channel error.
	5	EXTA3 channel error.
	6	EXTA1 channel error.
	7	Reserved bit
3	0	O2 signal <7mV during bench zero.
	1	O2 signal <3mV during bench zero.
	2	Reserved.
	3	Hexane span fail.
	4	Propane span fail.
	5	CO2 span fail.
	6	CO span fail.
	7	NO span fail.

$3F SET VACUUM INTERLOCK THRESHOLD LEVEL

The 7911 gas analyzer measures the vacuum pressure transducer 512 times per second in order to sense occlusions in the gas sampling lines. If the vacuum value exceeds a designated level, the EXTINT output signal (pin 6 on the 20 connector) is set high. This signal indicates a vacuum condition that exceeds the limit set and stored in EEPROM using the SET VACUUM INTERLOCK THRESHOLD command. If the vacuum signal drops below the threshold value, the EXTINT signal will be set low. Bit 4 of the status bit will be set true when the vacuum threshold is exceeded. In addition, the user control lines designated with the userer I/O mask will be set true when the vacuum threshold is exceeded. (see set *USER I/O MASK*). **The I/O lines are latched and do not reset to their default condition when the vacuum signal drops below the threshold value**.

**Command**	**$02<br>$3F<br>V1 V2<br>CS1 CS2**	**ASCII<br>ASCII<br>8 bit<br>Checksum**	**STX character<br>Command character<br>Threshold value<br>Checksum**
**Response**	**$02<br>$3F<br>V1 V2<br>ST1 ST2<br>CS1 CS2**	**ASCII<br>ASCII<br>8 bit<br>Status<br>Checksum**	**STX character<br>Command character<br>Value written to EEPROM<br>Status information<br>Checksum**
**Timeout**	**2 seconds**		

$40 ZERO FLOW CHANNEL

The pressure transducer used to measure the flow rate of the sampled gas can have a permanent voltage offset. This offset can be eliminated by issuing the *ZERO FLOW* command to the flow channel when the sampling pump is turned off.

**Command**	**$02<br>$40<br>$E4 $D0**	**ASCII<br>ASCII<br>Checksum**	**STX character<br>Command character<br>Checksum**
**Response**	**$02<br>$40<br>ST1 ST2<br>CS1 CS2**	**ASCII<br>ASCII<br>Status<br>Checksum**	**STX character<br>Command character<br>Status information<br>Checksum**
**Timeout**	**2 seconds**		

$41 SET USER CONTROL SIGNALS

User control lines can be used to turn on and off system components such as pneumatic valve solenoids and pump drive electronics. Eight digital control lines are available to the user for system controls. Each bit of the external control byte enables or disables one of the digital control lines. 1=ON 0=OFF.

**Command**	**$02**<br>**$41**<br>**V1 V2**<br>**CS1 CS2**	**ASCII**<br>**ASCII**<br>**8 bit**<br>**Checksum**	**STX character**<br>**Command character**<br>**Control bits**<br>**Checksum**
**Response**	**$02**<br>**$41**<br>**ST1 ST2**<br>**CS1 CS2**	**ASCII**<br>**ASCII**<br>**Status**<br>**Checksum**	**STX character**<br>**Command character**<br>**Status information**<br>**Checksum**
**Timeout**	**2 seconds**		

$42 ZERO O2

Due to the high gain of the oxygen channel, small voltage offsets in the amplifier circuits can cause an offset in the oxygen concentration. The ZERO O2 command is used to correct for these electronic offsets. Since the oxygen zero level is stored in EEPROM memory, this command is normally needed only once during the life of the product (typically during factory calibration). A zero signal can be created using sample gas with no oxygen present, or by shorting the O2 input pins. **This command should only be used with a zero oxygen signal.**

**Command**	**$02**<br>**$42**<br>**$E4 $D2**	**ASCII**<br>**ASCII**<br>**Checksum**	**STX character**<br>**Command character**<br>**Checksum**
**Response**	**$02**<br>**$42**<br>**ST1 ST2**<br>**CS1 CS2**	**ASCII**<br>**ASCII**<br>**Status**<br>**Checksum**	**STX character**<br>**Command character**<br>**Status information**<br>**Checksum**
**Timeout**	**2 seconds**		

$43 CLEAR LOW O2 STATUS BIT

The clear low O2 status bit is used after a new electrochemical O2 sensor is installed in the analyzer system.

**Command**	**$02**<br>**$43**<br>**$E4 $D3**	**ASCII**<br>**ASCII**<br>**Checksum**	**STX character**<br>**Command character**<br>**Checksum**
**Response**	**$02**<br>**$43**<br>**ST1 ST2**<br>**CS1 CS2**	**ASCII**<br>**ASCII**<br>**Status**<br>**Checksum**	**STX character**<br>**Command character**<br>**Status information**<br>**Checksum**
**Timeout**	**2 seconds**		

$44 CLEAR NO ERROR BIT

The NOx error bit (XERR2 bit 3) is set if the NOx input signal exceeds the data acquisition limit. This error bit can be cleared using the $44 command

**Command**	**$02**<br>**$44**<br>**$E4 $D4**	**ASCII**<br>**ASCII**<br>**Checksum**	**STX character**<br>**Command character**<br>**Checksum**
**Response**	**$02**<br>**$44**<br>**ST1 ST2**<br>**CS1 CS2**	**ASCII**<br>**ASCII**<br>**Status**<br>**Checksum**	**STX character**<br>**Command character**<br>**Status information**<br>**Checksum**
**Timeout**	**2 seconds**		

$45 SET USER CONTROL MASK

If the vacuum threshold is exceeded, the bench embedded software sets each user control line that is designated in the USER CONTROL MASK. The USER CONTROL MASK is an 8 bit number that forms the bit configuration for the control lines. Bit0 corresponds to User0, Bit1 corresponds to User1, etc. On power up and after a RESET command the control signal interlock state is set to $00. The USER CONTROL MASK value is not stored in EEPROM. It is therefore necessary to configure this mask during system initialization.

The control signal interlock configuration byte is usually set to a “safe” condition during normal operation to guard against sudden water ingress into the analyzer. During leak checking, the control mask value must be changed to allow vacuum conditions used in the leak check procedure. The mask should be reset to its original value when leak check operations are completed.

Control lines are set true within 2 msec from the time the vacuum signal reaches the threshold vacuum. Further details are described in the *SET VACUUM THRESHOLD* command section.

**Command**	**$02**<br>**$45**<br>**V1 V2**<br>**CS1 CS2**	**ASCII**<br>**ASCII**<br>**8 bit**<br>**Checksum**	**STX character**<br>**Command character**<br>**Control line config.**<br>**Checksum**
**Response**	**$02**<br>**$45**<br>**ST1 ST2**<br>**CS1 CS2**	**ASCII**<br>**ASCII**<br>**Status**<br>**Checksum**	**STX character**<br>**Command character**<br>**Status information**<br>**Checksum**
**Timeout**	**2 seconds**		

$46 READ CONTROL LINE STATE

Used to read the state of the user control lines.

**Command**	**$02**<br>**$46**<br>**$E4 $D6**	**ASCII**<br>**ASCII**<br>**Checksum**	**STX character**<br>**Command character**<br>**Checksum**
**Response**	**$02**<br>**$46**<br>**IO1 IO2**<br>**ST1 ST2**<br>**CS1 CS2**	**ASCII**<br>**ASCII**<br>**8 bit**<br>**Status**<br>**Checksum**	**STX character**<br>**Command character**<br>**Control line status**<br>**Status information**<br>**Checksum**
**Timeout**	**2 seconds**		

$47 ZERO REQUEST INTERVAL

The 7911 bench requests a zero command periodically to maintain full accuracy. The zero request schedule is as follows:

1. A zero request (bit 1 of the status byte is true) is set at power on. A zero operation should be performed as soon as possible after power on.

2. If a zero command is performed and the time after power on is:
   a) > 15 minutes next zero request = 12 minutes.
   b) > 8 minutes next zero request = 7 minutes.
   c) > 3 minutes next zero request = 5 minutes.
   d) < 3 minutes next zero request = 3 minutes.

This command allows the interval between zero requests, after 15 minutes from power on to be changed from the default value of 12 min. Time is specified in 0.5 second increments. Since the value is limited by a 16 bit value, the maximum time interval without a zero request is 32768 seconds or 9.1 hours. More frequent zero operations are recommended.

**Command**	**$02<br>$47<br>T1 T2 T3 T4<br>CS1 CS2**	**ASCII<br>ASCII<br>16 bit<br>Checksum**	**STX character<br>Command character<br>Zero request interval<br>Checksum**
**Response**	**$02<br>$47<br>ST1 ST2<br>CS1 CS2**	**ASCII<br>ASCII<br>Status<br>Checksum**	**STX character<br>Command character<br>Status information<br>Checksum**
**Timeout**	**2 seconds**		

$48 READ BENCH ID

Crestline Instruments has developed several automotive gas analyzers with different levels of product capabilities. The bench ID command allows the host system to identify which bench product is present in the system.

7911 I.D. = 03

IRidium100 and IRidium50 are registered trademarks of Honeywell Corporation.

**Command**	**$02<br>$48<br>$E4 $D8**	**ASCII<br>ASCII<br>Checksum**	**STX character<br>Command character<br>Checksum**
**Response**	**$02<br>$48**	**ASCII<br>ASCII**	**STX character<br>Command character**

	**ID**<br>**ST1 ST2**<br>**CS1 CS2**	**ASCII**<br>**Status**<br>**Checksum**	**Bench I.D. code**<br>**Status information**<br>**Checksum**
**Timeout**	**2 seconds**		

$49 LOAD PROGRAM INTO FLASH MEMORY

The 7911 analyzer uses a microprocessor with flash memory. Flash memory provides the capability to change the system software if new versions become available. The $49 command provides a convenient means for updating software. When the $49 command is sent to the bench, the bench embedded system branches to a software loading routine. This routine erases the previous software version, reads **encrypted Intel .HEX files**, performs checksum verification from the data received, and validates the values written to flash memory. If an error is detected, the loader will terminate. The analyzer will enter this routine on power up until a successful download is completed. **Only software distributed by Crestline Instruments should be used for program updates. Non encrypted files downloaded to the analyzer will fail to operate.**

**Command**	**$02**<br>**$49**<br>**$E4 $D9**	**ASCII**<br>**ASCII**<br>**Checksum**	**STX character**<br>**Command character**<br>**Checksum**
**Response**	**$02**<br>**$49**<br>**ST1 ST2**<br>**CS1 CS2**	**ASCII**<br>**ASCII**<br>**Status**<br>**Checksum**	**STX character**<br>**Command character**<br>**Status information**<br>**Checksum**
**Timeout**	**2 seconds**		

$4A Bench Operating Status Bits

Four status bytes are available to query the operating conditions of the bench. All bits are reset at power on and during the execution of the RESET command.

**Command**	**$02**<br>**$4A**<br>**$E4 $DA**	**ASCII**<br>**ASCII**<br>**8 bit Checksum**	**STX character**<br>**Command character**<br>**Checksum**
**Response**	**$02**<br>**$4A**<br>**STATUS0**<br>**STATUS1**<br>**STATUS2**<br>**ST1 ST2**<br>**CS1 CS2**	**ASCII**<br>**ASCII**<br>**8 bit**<br>**8 bit**<br>**8 bit**<br>**Status**<br>**Checksum**	**STX character**<br>**Command character**<br>**Operating status**<br>**Operating status**<br>**Operating status**<br>**Normal Status byte**<br>**Checksum**
**Timeout**	**2 seconds**		

Status Byte Definitions

**Byte**	**Bit**	**Meaning**	**State at Power On**
**0**	**0**	1 = Temperature out of operating range.	**0**
	**1**	1 = Pressure out of operating range.	**0**
	**2**	1 = Reference signal is too low—any time.	**0**
	**3**	1 = HC Signal is too low during zero command.	**0**
	**4**	1 = CO signal is too low during zero command.	**0**
	**5**	1 = CO2 signal is too low during zero command.	**0**
	**6**	1 = O2 signal is less than 5mV during zero command.	**0**
	**7**	1 = NO signal is too low. Currently level is not defined.	**0**
**1**	**0**	1 = Hexane span fail. Cleared with successful span or span reset.	**Last span result**
	**1**	1 = Propane span fail. Cleared with successful span or span reset.	**Last span result**
	**2**	1 = CO span fail. Cleared with successful span or span reset.	**Last span result**
	**3**	1 = CO2 span fail. Cleared with successful span or span reset.	**Last span result**
	**4**	1 = NO span fail. Cleared with successful span or span reset.	**Last span result**
	**5**	1 = O2 span fail.	**Last span result**
	**6**	1 = ZERO or SPAN in progress.	**0**
	**7**	1 = Warmup mode. 0 = Normal operating mode.	**1**
**2**	**0**	User control line 0.	**0**
	**1**	User control line 1.	**0**
	**2**	User control line 2.	**0**
	**3**	User control line 3.	**0**
	**4**	User control line 4.	**0**
	**5**	User control line 5.	**0**
	**6**	User control line 6.	**0**
	**7**	User control line 7.	**0**
			**0**

$4B Extended Compensated Data

A new command has been created that combines all compensated data into one command. This eliminates the need to alternate between the original compensated data command and the environmental command. Vacuum, pressure, and user ADC signals are now provided as 16 bit data to take advantage of the 10 bit resolution from the microprocessor ADC. The status bytes described in command $4A are also included.

**Command**	**$02**<br>**$4B**<br>**$E4 $DB**	**ASCII**<br>**ASCII**<br>**8 bit Checksum**	**STX character**<br>**Command character**<br>**Checksum**
**Response**	**$02**<br>**$4B**<br>**hex1 hex2 hex3 hex4**<br>**pro1 pro2 pro3 pro4**<br>**CO21 CO22 CO23 CO24**<br>**CO1 CO2 CO3 CO4**<br>**O21 O22 O23 O24**<br>**NOx1 NOx2 NOx3 NOx4**<br>**Tach6 Tach5 Tach4 Tach3 Tach2 Tach1**<br>**Pres1 Pres2 Pres3 Pres4**<br>**Tmp1 Tmp2 Tmp3 Tmp4**<br>**Vac1 Vac2 Vac3 Vac4**<br>**ADC11 ADC12 ADC13 ADC14**<br>**ADC21 ADC22 ADC23 ADC24**<br>**ADC31 ADC32 ADC33 ADC34**<br>**Stat11 Stat12**<br>**Stat21 Stat22**<br>**Stat31 Stat32**<br>**STATUS1 STATUS2**<br>**CS1 CS2**	**ASCII**<br>**ASCII**<br>**16 bit**<br>**16 bit**<br>**16 bit**<br>**16 bit**<br>**16 bit**<br>**16 bit**<br>**24 bit**<br><br>**16 bit**<br>**16 bit**<br>**16 bit**<br>**16 bit**<br>**16 bit**<br>**16 bit**<br>**8 bit**<br>**8 bit**<br>**8 bit**<br>**Status**<br>**Checksum**	**STX character**<br>**Command character**<br>**Ppm hexane**<br>**Ppm propane**<br>**Vol % CO2 x 100**<br>**Vol % CO x 1000**<br>**Vol % O2 x 100**<br>**Ppm Nitric oxide**<br>**Tachometer time interval sec x 2,000,000**<br>**Pressure (Kpa)**<br>**Temperature (deg K)**<br>**Vac pressure (Kpa)**<br>**User signal (volts)**<br>**User signal (volts)**<br>**User signal (volts)**<br>**Operating status**<br>**Operating status**<br>**Operating status**<br>**Normal Status byte**<br>**Checksum**
**Timeout**	**2 seconds**		

$4C O2 SENSOR FAILURE LIMIT

This command sets the voltage level where the O2 fail bit is set. The level byte represents 25.6 mV. One count therefore corresponds to 0.1mV. 50 counts would be 5mV. The default value from the factory is 50 or 5 mV.

**Command**	**$02**<br>**$4C**<br>**V1 V2**<br>**V1 V2**<br>**CS1 CS2**	**ASCII**<br>**ASCII**<br>**8 bit warning value**<br>**8 bit error value**<br>**Checksum**	**STX character**<br>**Command character**<br>**mV = 0.1 * value**<br>**mV = 0.1 * value**<br>**Checksum**
**Response**	**$02**<br>**$4C**<br>**ST1 ST2**<br>**CS1 CS2**	**ASCII**<br>**ASCII**<br>**Status**<br>**Checksum**	**STX character**<br>**Command character**<br>**Status information**<br>**Checksum**
**Timeout**	**2 seconds**		

$4D METHANE/PROPANE MODE CONTROL

This command is used to set one of two modes whereby the normal propane data can be changed to methane. In the command format shown below M1 and M2 represent an eight bit byte that designates either methane or propane mode.

Mode = 0000 propane mode
Mode 0001 methane mode

The mode must be set by the host computer each time the bench is powered up since the mode information is stored in RAM. **The reset command destroys the state of the mode control. If the reset command is used the mode command should follow to assure that the bench is operating in the proper mode.** There is no status bit to indicate in which mode the bench is operating. The host program must manage the mode state.

Use of the mode command:

When the mode is set to propane (the default) the bench operates normally. See this manual for details. If the bench is in the methane mode , the following changes are invoked:

1. The COMPENSATED DATA command ($31) reports methane instead of propane. The concentration range is 0-65536 ppm methane. Due to the 16 bit limitation of the data format, negative results are reported as their absolute value.
2. The EXTENDED DATA command ($4B) reports methane instead of propane. The concentration range is 0-65536 ppm methane. Due to the 16 bit limitation of the data format, negative results are reported as their absolute value.

3. The mask byte in the SPAN command ($36) normally used for propane will designate a methane span request.

4. The mask byte in the SPAN RESET command ($38) normally used for propane will designate a methane span request.

5. If a span error occurs while spanning methane, the unused bit 2 in the service bytes will indicate a methane span error.

**Command**	**$02**<br>**$4D**<br>**M1 M2**<br>**CS1 CS2**	**ASCII**<br>**ASCII**<br>**8 bit**<br>**Checksum**	**STX character**<br>**Command character**<br>**Mode**<br>**Checksum**
**Response**	**$02**<br>**$4D**<br>**ST1 ST2**<br>**CS1 CS2**	**ASCII**<br>**ASCII**<br>**STATUS**<br>**Checksum+**	**STX character**<br>**Command character**<br>**STATUS**<br>**Checksum**
**Timeout**	**2 seconds**		

$4E Lamp on/off

This command enables the bench to be set in a standby mode where the bench is powered but the lamp is turned off in order to conserve power and extend lamp life. Several gas analyzers employ a similar strategy of turning off the pump in order to extend pump life when the analyzer is not performing active measurements. The command format is:
0x02 0x4E 0x80 0x80 CS1 CS2 to turn the lamp off
0x02 0x4E 0x80 0x81 CS1 CS2 to turn the lamp on

When the lamp off command is applied the following are invoked:

1. All commands except lamp on and the operating status command will be NAKed when the lamp is off.
2. The warmup bit is set when the lamp is off and is turned on 10 seconds after the lamp is turned on.
3. The power up timer is reset when the lamp is turned on . This resets the zero interval requests in accordance with power on.

**Command**	**$02**<br>**$4E**<br>**M1 M2**<br>**CS1 CS2**	**ASCII**<br>**ASCII**<br>**8 bit**<br>**Checksum**	**STX character**<br>**Command character**<br>**Mode**<br>**Checksum**
**Response**	**$02**<br>**$4E**<br>**ST1 ST2**<br>**CS1 CS2**	**ASCII**<br>**ASCII**<br>**Status**<br>**Checksum**	**STX character**<br>**Command character**<br>**Status information**<br>**Checksum**
**Timeout**	**2 seconds**		

4. $15 ERROR RESPONSE TO HOST MESSAGES (NAK)

If the 7911 bench can not interpret a host command, an illegal command is sent, or invalid parameters are used, then a “No Acknowledgment” message will be returned. This error message is formatted as follows:

Error response: $02 $15 ST1 ST2 CS1 CS2

The first character is the STX start of message character. This character is followed by the ASCII NAK code $15. The NAK code is followed by the analyzer status information. The message is finally terminated with the checksum characters.

CALCULATING CHECKSUMS

Checksum calculations for host commands:

The proposed analyzer calculates a checksum for each command received. This checksum is compared with the checksum supplied by the host. If the calculated checksum, and the supplied checksum do not agree, an error condition is set in the analyzer status word, and an error message is returned to the host.

The checksum is computed starting with the command character (The STX character is not included in the checksum calculation), and ends with the last data byte. The binary sum of all characters in this interval produces the checksum result. Overflow from an 8 bit byte is discarded producing an 8 bit checksum result. This binary data is converted to two hexadecimal digits. Each digit is transmitted as a separate byte according to the communications protocol where the most significant digit contains $E in the high order nibble and the least significant digit contains $D in the high order nibble.

Example: Given a checksum value of 4204 decimal. Binary = 0001 0000 0110 1100

The value of 4204 is converted to hexadecimal yielding hexadecimal 106C. **6C** is retained as the checksum. These two digits are transmitted in separate bytes as follows:

The high order nibble of the most significant byte = $E. The most significant byte would therefore be = $E6.

The high order nibble of the least significant byte = $D. The least significant byte would therefore be = $DC. The checksum is therefore transmitted as $E6 $DC.

Status Data

The 7911 bench provides status information in each data packet sent to the host system. This status information can be used to determine if the 7911 bench is operating correctly, or if it is being used incorrectly. The analyzer firmware contains an 8 bit status byte which stores the status of the following conditions:

**Bit 0**	Concentration out of range. If the concentration reported by the analyzer is negative or exceeds the maximum specification value, this bit is set.
**Bit 1**	Zero operation requested. To achieve optimum accuracy, the 7911 bench periodically issues<br>zero requests. These zero requests are issued at power up, at specific time intervals, or if the<br>temperature since the last zero changes by more than 3 degrees Celcius.
**Bit 2**	A command was received that could not be interpreted reliably. This is due to a non-existent command character or checksum (termination) character.
**Bit 3**	Checksum error. The calculated checksum does not match the transmitted value.
**Bit 4**	Product specifications violated. The analyzer checks the temperature, pressure, vacuum threshold exceeded and PEF values. If any of these parameters are outside the product specifications this bit is set. This bit is not latched and will clear if the violated parameter returns to conforming limits.
**Bit 5**	EEPROM address out of range.
**Bit 6**	Infrared signal low. If the infrared source or the electronics used to detected the infrared signal degrades to a preset level, this bit is set.
**Bit 7**	Hardware fault. Service required. The bench failed due to:<br>1. RAM test fail.<br>2. ROM checksum error.<br>3. Low light signal on HC, CO2 or CO channels during zero.<br>4. Low light signal on reference channel any time.<br>5. Signals out of range of A/D converter.<br>6. EEPROM write error.
